# Differential Binding of NLRP3 to non-oxidized and Ox-mtDNA mediates NLRP3 Inflammasome Activation

**DOI:** 10.1038/s42003-023-04817-y

**Published:** 2023-05-30

**Authors:** Angela Cabral, Julia Elise Cabral, Angelina Wang, Yiyang Zhang, Hailin Liang, Donya Nikbakht, Leslie Corona, Hal M. Hoffman, Reginald McNulty

**Affiliations:** 1 Department of Molecular Biology and Biochemistry, University of California Irvine, Steinhaus Hall, Irvine, CA 92694-3900 USA; 2 Division of Pediatric Allergy, Immunology, and Rheumatology, Rady Children’s Hospital of San Diego, University of California, San Diego, San Diego, CA USA

**Keywords:** Innate immunity, Structural biology

## Abstract

The NLRP3 inflammasome is a key mediator of the innate immune response to sterile tissue injury and is involved in many chronic and acute diseases. Physically and chemically diverse agents activate the NLRP3 inflammasome. Here, we show that NLRP3 binds non-oxidized and Ox-mtDNA differentially, with a half maximum inhibitory concentration (IC_50_) for non-oxidized and Ox-mtDNA of 4 nM and 247.2 nM, respectively. The NLRP3 Neonatal-Onset Multisystem Inflammatory Disease (NOMID) gain of function mutant could bind non-oxidized mtDNA but had higher affinity for Ox-mtDNA compared to WT with an IC_50_ of 8.1 nM. NLRP3 lacking the pyrin domain can bind both oxidized and non-oxidized mtDNA. Isolated pyrin domain prefers Ox-mtDNA. The NLRP3 pyrin domain shares a protein fold with DNA glycosylases and generate a model for DNA binding based on the structure and sequence alignment to *Clostridium acetobutylicum* and human OGG1, an inhibitor of Ox-mtDNA generation, 8-oxoguanine DNA glycosylases. We provide a new model for how NLRP3 interacts with Ox-mtDNA supported by DNA binding in the presence of a monoclonal antibody against the pyrin domain. These results give new insights into the mechanism of inflammasome assembly, and into the function of reactive oxygen species in establishing a robust immune response.

## Abstract (web summary)

The differential binding of NLRP3 to non-oxidized and Ox-mtDNA is characterized, which provides insight into the mechanisms of inflammasome assembly

## Introduction

The innate immune system recognizes pathogen-associated molecular patterns (PAMP) using Toll-like receptors (TLR’s) located in mammalian membranes. Bacterial debris containing lipopolysaccharide (LPS) binding TLR4 causes an NF-kB-dependent expression of key genes in innate immunity and inflammation. The Nucleotide oligomerization domain (NOD)-like receptor pyrin domain containing 3 (NLRP3) is an NF-kB induced sensor protein that mediates inflammasome activation and production of the biologically active form of cytokine IL-1β, in response to sterile tissue injury^1,2^. IL-1β is secreted and signals to surrounding cells to produce additional cytokines. Gasdermin D, which is processed by inflammasome-activated Caspase-1, is inserted into the membrane, causing leakage of intracellular contents^3^. In addition to NLRP3 consisting of a pyrin, NOD or NACHT, and leucine-rich repeat (LRR) domains, the NLRP3 inflammasome contains three other subunits, ASC, NEK7, and Caspase-1. Human NLRP3 oligomerization results in a decamer formed by two back-to-back pentamers^4^. Activation-related conformational change allows NLRP3 to bind ASC and activate Caspase-1, a cysteine protease that cleaves pro-IL-1β cytokine to yield a secretion-enabled IL-1β^5^.

Macrophages require a two-step mechanism, priming and activation for NLRP3 inflammasome-induced production of IL-1β^6^. Priming involves TLR-signaling, which leads to NF-kB activation and transcription of the NLRP3 and IL-1β genes^7^. Activation occurs with diverse agents, including bacterial toxins, microcrystalline substances, ATP, monosodium urate (MSU), calcium pyrophosphate dihydrate (CPPD), silica, asbestos, alum, and hydroxyapatite (HA). Humans harboring one of many NLRP3 point mutations^8^ suffer from diseases that result in Familial cold-induced autoinflammatory syndrome (FCAS), Muckle-Wells Syndrome (MWS), Chronic infantile neurologic cutaneous articular (CINCA) syndrome/Neonatal-onset multisystem inflammatory disease (NOMID), which are collectively called cryopyrin-associated periodic syndromes (CAPS) due to constitutively active NLRP3^9^, are more sensitive to activating stimuli. Since none of the agents that activate NLRP3 were shown to bind NLRP3 and are very dissimilar in structure, it is suggested that they trigger a common cellular event that leads to NLRP3 activation. One such common event is the production of oxidized (Ox) mitochondrial (mt) DNA^10^.

Reactive oxygen species (ROS) generated during activation-induced mt damage react with newly synthesized mtDNA to generate Ox-mtDNA that can be crosslinked and precipitated with NLRP3^10,11^, but direct interaction with NLRP3 remains to be demonstrated. In this paper, we use both Ox and non-oxidized mt D-loop DNA under native conditions with pulldown and EMSA experiments to show that a complex is formed between NLRP3 and mtDNA with a preference for Ox-mtDNA. We also propose a new model of how NLRP3 interacts with Ox-mtDNA based on homology modeling to glycosylases which are known to interact with ox-DNA^12,13^, including OGG1, which prevent Ox-mtDNA generation and NLRP3 inflammasome activation^14^. These results provide important new insights into the process whereby Ox-mtDNA activates the NLRP3 inflammasome.

## Results

### EMSA of NLRP3 with oxidized DNA

**Fig. 1:**
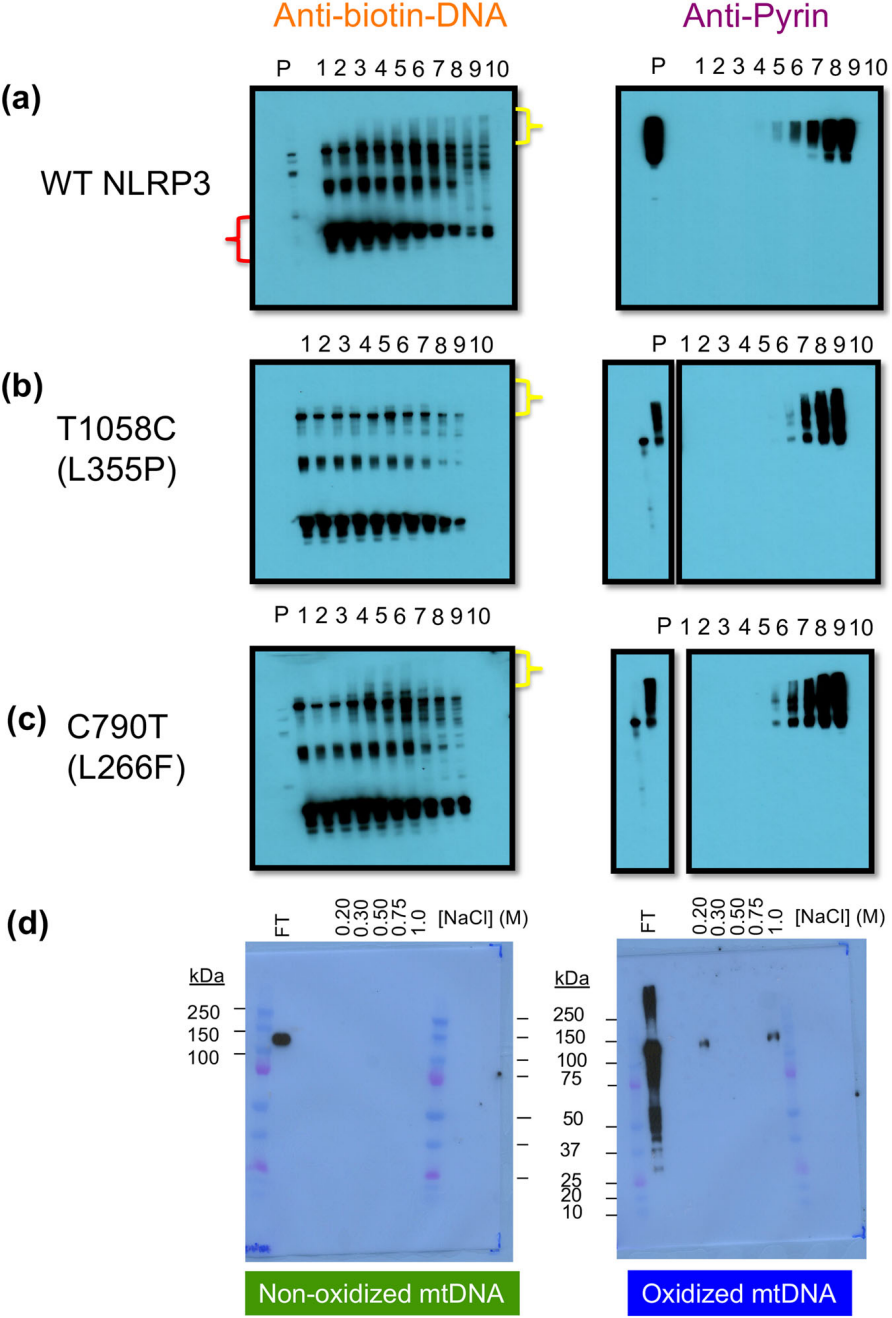
NLRP3 binds oxidized mtDNA. Constant ox-mtDNA concentrations were incubated with increasing 1:2 dilutions of protein extract (lanes numbered 1-low [protein] to 10 high [protein]). Lane P is protein with mtDNA. **a** Left panel -Free Ox-mtDNA (red bracket) decreases with increasing NLRP3 extract (left to right). The oxidized mtDNA shifts from free to protein-bound (yellow bracket). Right panel- Gel reprobed for NLRP3 shows the same localization and pattern of shift. **b** No shift observed with NLRP3 FCAS mutation T1058C (L355P) (yellow bracket) or **c** NOMID mutation C790T (L266F). **d** Pulldown of NLRP3 to magnetic Dynabeads coated in either non-ox-DNA (left) or ox-DNA (right). Elution was performed by increasing [NaCl] from 0.2 to 1 M.

Since previous studies show that ox-DNA can activate NLRP3 in macrophages^10^, we tested if NLRP3 expressed in human Expi293 cells could, in fact, bind ox-DNA. We transfected Expi293 cells with mammalian expression vectors containing wild-type NLRP3 or point mutant versions found in CAPS patients. Cells were harvested after allowing protein expression for 3 days. The electromobility shift assay (EMSA) was used to assess the shift of ox-DNA in the presence of increasing concentrations of NLRP3-containing extract (Fig. 1). Using a constant concentration of biotinylated ox-DNA, increasing concentrations of wild-type NLRP3 caused a gradual shift in ox-DNA migration (Fig. 1a). Although there were other proteins in the gel extract that also bound the ox-DNA, there was a notable shift that clearly increased with protein concentration in the same vicinity where NLRP3 migrates. Moreover, the increased intensity of DNA migrating at the top of the gel was coupled to a loss of free DNA at the bottom of the gel (Fig. 1a). To examine if this shift in ox-DNA was localized with NLRP3, the membrane was stripped and reprobed with a monoclonal antibody for NLRP3 (Fig. 1a). The increasing concentration of NLRP3 shown with the NLRP3 antibody matched the increase in shifted ox-DNA. As expected, NLRP3 was not detected in non-transfected cells since these cells have no detectable expression of NLRP3 (Supplementary Fig. 1). Non-transfected cells showed non-specific binding of ox-DNA, but not with the same increasing pattern localized with NLRP3 (Supplementary Fig. 2). Two mutants representative of those found in CAPS patients showed a different binding pattern with ox-DNA (Fig. 1b, c). FCAS mutant T1058C (L355P) had no detectible shift in binding ox-DNA in the NLRP3 region of the membrane (Fig. 1b), while the NOMID mutant C790T (L266F) showed a shift, but not at the highest protein concentration of NLRP3 (Fig. 1c). In this case, there is a shift in the upper third of the gel, but not in the vicinity where NLRP3 resides. NLRP3 was localized roughly in the same area of the gel regardless of binding to DNA. The NLRP3 CAPS mutants’ lack of shift suggests there is no binding or that binding has occurred and the complex did not enter the gel, or transferred differentially from the majority of other proteins in the lysate.

### High stringency elutes NLRP3 from oxidized DNA

The EMSA showed that NLRP3-containing cell extract could co-localize with ox-DNA; however, the possibility remained that the NLRP3 complex was not playing any role in binding ox-mtDNA, even though it co-localized with the shift. The shift could be caused by a protein of similar size in the same vicinity of the membrane. To test the hypothesis that NLRP3 could directly bind ox-mtDNA, we developed a pulldown assay. Briefly, magnetic streptavidin Dynabeads were incubated with either biotinylated ox-DNA or biotinylated non-ox-mtDNA. Both sets of beads were washed to remove unbound DNA, then exposed to purified NLRP3 extract. After washing, beads were treated with increasing concentrations of NaCl stepwise to elute NLRP3. Beads pretreated with ox-mtDNA did show NLRP3 elution with a low salt wash of 0.2 M NaCl. NLRP3 did not elute with 0.3–0.75 M NaCl. NLRP3 protein was eluted from the oxidized mtDNA coated beads with 1 M NaCl (Fig. 1d). The elution with low salt was not expected but could be due to a smaller NLRP3 complex (or monomer) that has a lower amount of surface charge. NLRP3 could not be detected eluting from non-oxidized beads (Fig. 1d).

### Elution of NLRP3 with mitochondrial DNA

**Fig. 2:**
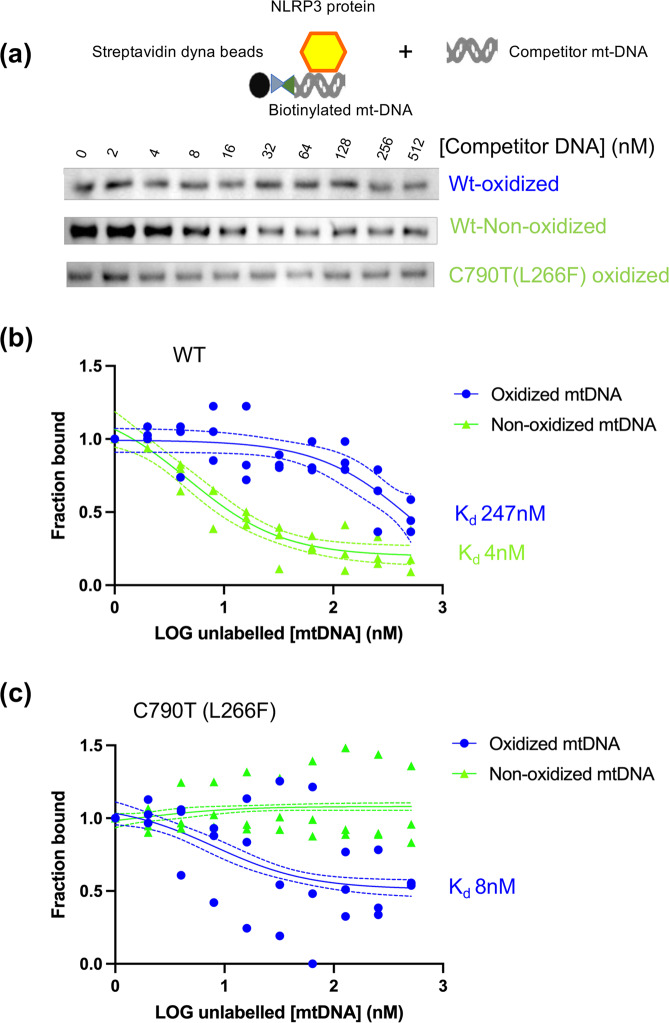
NLRP3 elutes with unlabeled oxidized and non-oxidized competitor mtDNA. **a** NLRP3 was eluted from biotinylated oxidized or non-oxidized mtDNA coated streptavidin Dynabeads with increasing concentrations of unlabeled oxidized or non-oxidized mtDNA (schematic). A representative gel of each membrane of each shown (*n* = 3). **b**, **c** Fraction bound vs. LOG unlabeled mtDNA for elution with oxidized or non-oxidized mtDNA. Raw data plotted. The dashed line represents a 95% confidence interval of best-fit line (solid line), *n* = 3.

Since increasing concentrations of salt enabled NLRP3 elution from ox-DNA-containing Dynabeads, it seemed logical that mitochondrial DNA could be used to elute NLRP3 in the same fashion. Moreover, we also wanted to examine the bound fraction to detect NLRP3 since failure to detect in non-oxidized mtDNA eluate could be because the protein was still bound to the beads and not due to lack of binding (Fig. 1d). To test this hypothesis, we incubated streptavidin beads with biotinylated oxidized or non-oxidized mtDNA. Following wash steps to remove unbound DNA, NLRP3 protein was added to the beads and subsequently treated with increasing concentrations, separately, of unlabeled competitor oxidized or non-oxidized mtDNA (Fig. 2a). Increasing concentrations of DNA resulted in greater amounts of NLRP3 eluted from the beads when visualized via western blot with an NLRP3 pyrin antibody (Fig. 2a). The gel bands were quantified with Image J and the fraction bound was plotted with GraphPad Prism software to determine a half maximal inhibitory concentration (IC_50_). Non-linear regression analysis for wild-type NLRP3 with non-oxidized mtDNA revealed an R-squared of 0.98 with a calculated IC_50_ of 4.8 nM. The 95% Cl (profile likelihood) for the IC_50_ was 2.6-8.4 nM. (Fig. 2b). However, the wild-type NLRP3 interaction with oxidized mtDNA had a much poorer non-linear regression fit with an R-squared of 0.6 and calculated IC_50_ of 247 nM. This indicates NLRP3 interacts differentially with oxidized and non-ox-DNA.

As the NLRP3 C790T (L266F) mutation represents a gain of function mutation found in patients with NOMID, we investigated its interaction with oxidized and non-oxidized mtDNA. The C790T (L266F) mutant had a higher affinity for oxidized mtDNA with a calculated IC_50_ of 8.1 nM compared to the wild type (247 nM). The 95% Cl profile likelihood was between 3.5–18.1 nM. The interaction with non-ox-DNA had a poor fit with non-linear regression analysis with an R-squared of 0.73 (Fig. 2c).

### Analysis of structure for mtDNA binding location

**Fig. 3:**
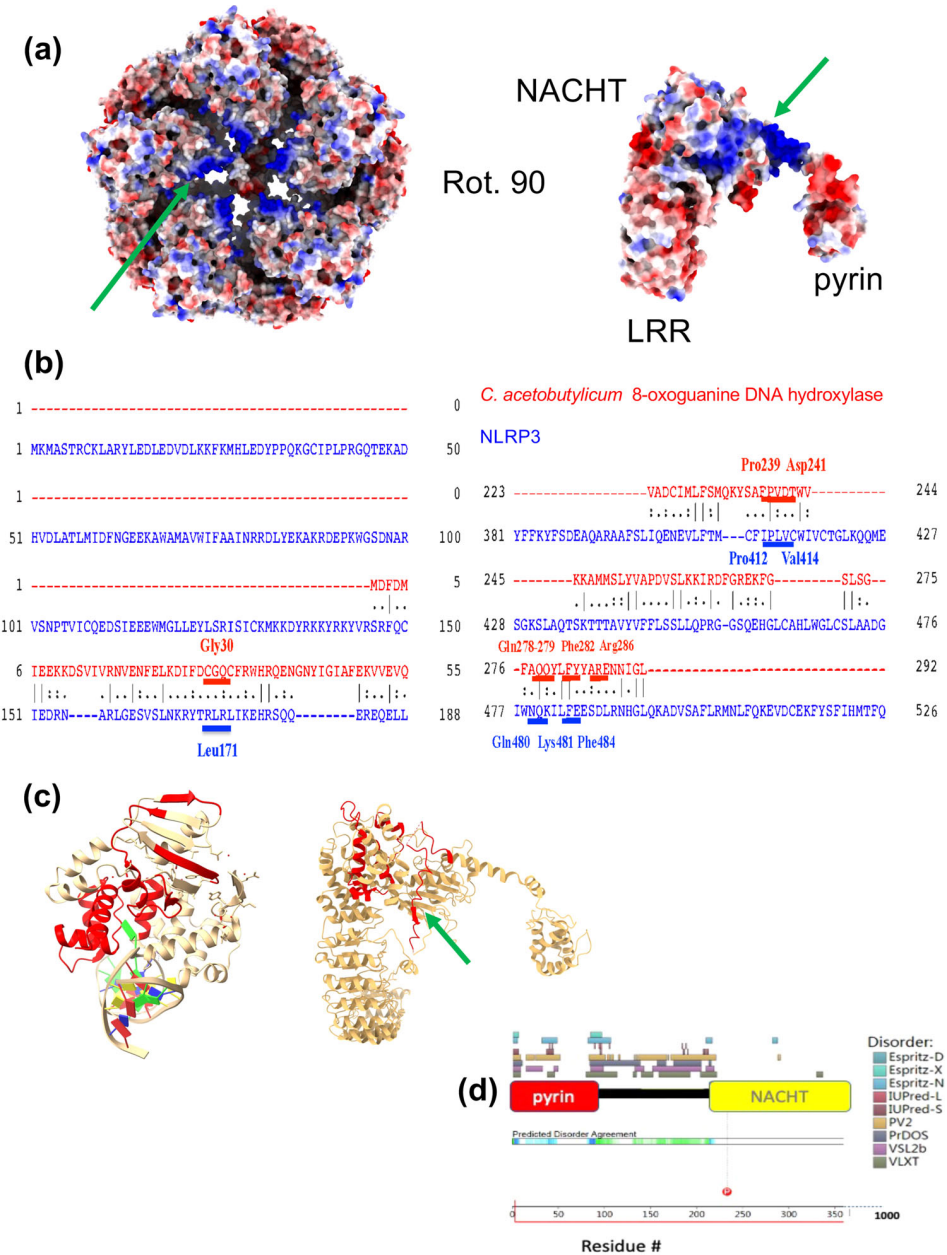
Positive patch in the intrinsically disordered region shared with CacOgg. **a** NLRP3 decamer (top) and NLRP3 molecule A (bottom) from PDBID 7pzc. Electrostatic surface potential illustrates the putative DNA binding site in the large positive patch (green arrow). **b** Sequence alignment of NLRP3 and *C. acetobutylicum* 8-oxoguanine DNA hydroxylase. Residues important in binding ox-DNA for CacOgg are shown in red. Corresponding residues for NLRP3 are shown in blue. The three-letter amino acid code for residues demonstrated to interact with ox-DNA shown for glycosylase (red) and corresponding for NLRP3 (blue). **c** Ribbon diagram of CacOgg bound to ox-DNA (left) (protein databank code-3FOZ) and NLRP3 (right) with aligned residues for each protein (red). **d** The sequence between pyrin and NACHT domains are predicted to contain several intrinsically disordered regions. For simplicity, the rest of the protein containing the LRR domain is not shown. Results from disorder predictor programs are superposed above, color-coded with the key on the right. Intrinsically disordered region (IDR) mapped to NLRP3 3D structure (green arrow in (**c**)).

We investigated the cryo-EM structure of full-length human NLRP3^4^ to search for putative mtDNA binding sites. A view of the decamer along the pseudo fivefold axis (top view) shows a starfish-shaped solvent-accessible surface area with a high positive charge localized to the interior region (Fig. 3a). This solvent-accessible gap traverses through the entire decamer. Electrostatic surface potential calculation illustrates a positive charge localized to the medial portion of the starfished-shaped pore (Fig. 3a). A single subunit of the decamer shows the large positive surface spans from the NACHT domain and is continuous through the linker region, which is between the NACHT and pyrin domains (Fig. 3a).

### Intrinsic disorder between pyrin and NACHT Domains is shared with CacOgg

Since we showed NLRP3 can bind oxidized and non-oxidized mtDNA, and the structure illustrates putative regions for binding, we searched for proteins that are known to bind ox-DNA and 8-oxo-dGTP nucleosides. We performed pairwise alignment of NLRP3 with bacterial and human glycosylase using EMBOSS Needle. Although we could not find high sequence alignment for the entire protein, we found several stretches of residues that were exactly the same or very similar as with the *C. acetobutylicum* glycosylase (PDB 3F0Z) also called CacOgg (Fig. 3b). We noted the residues known to be important in binding ox-DNA for the glycosylase that were exactly the same as NLRP3 were Pro239, Gln278, Phe282 (CacOgg)^12^ and Pro412, Gln480, Phe484 (NLRP3) (Fig. 3b). The pairwise alignment was mapped to the 3D structure to see corresponding residues between CacOgg bound to ox-DNA (PDB 3I0W) and NLRP3 (Fig. 3c). The long stretch of positive surface (Fig. 3a) localizes to a shared region with CacOgg (Fig. 3c). Since this region appears mostly disordered in NLRP3 and not completely resolved in the NLRP3 decamer (Fig. 3a, c), we analyzed if NLRP3 fits the conical definition of an intrinsically disordered protein (IDP). Intrinsically disordered regions (IDR’s) are oftentimes known to convert from disordered to ordered, or vice versa, when interacting with macromolecules^15^. Software D^2^P^2^ ^16^ analysis showed that the 100-residue stretch between NLRP3 pyrin and NACHT domains is an IDR (Fig. 3d). Most of the predicted disorder agreement spans from half the pyrin-NACHT linker region all the way up to the NACHT domain. Other NLR’s that had similar IDR’s included NLRP 6, 10, and 12 (Supplementary Fig. 3).

### NLRP3 pyrin domain shares protein fold with human glycosylase

**Fig. 4:**
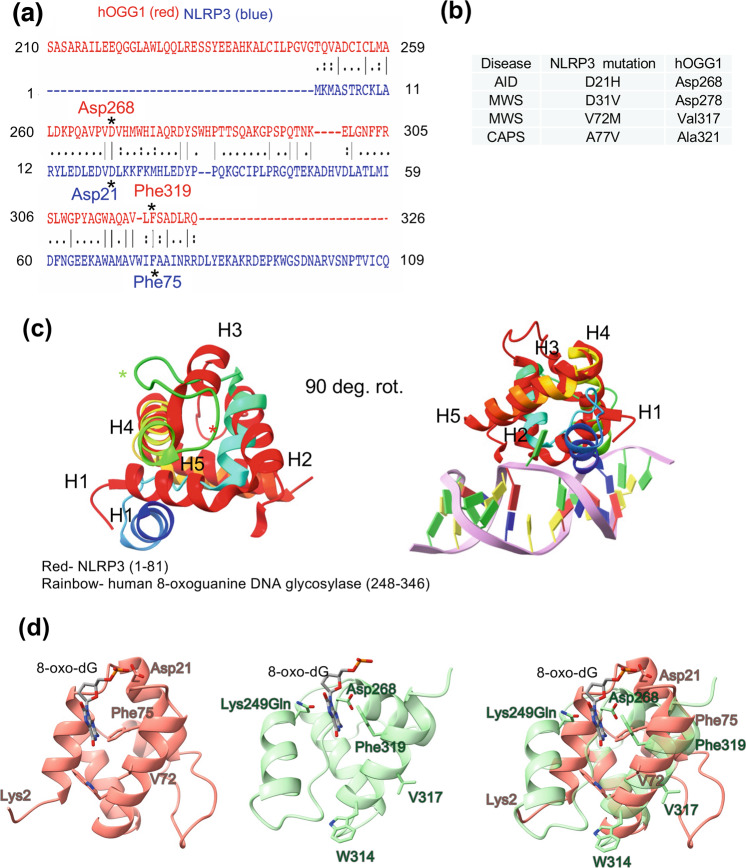
NLRP3 pyrin domain shares fold with human DNA glycosylase. **a** Sequence alignment of NLRP3 with human 8-oxoguanine DNA glycosylase (hogg1). Critical amino acids for hogg1 that bind ox-DNA are shown in red and corresponding residues for NLRP3 are in blue. **b** NLRP3 disease mutations in the pyrin domain and corresponding residues for human glycosylase (hogg1). **c** Left- topology and fold of NLRP3 (red) for pyrin residues 1–81 superposed with hogg1 bound to ox-DNA (rainbow). From N- to C-terminus, helices 1–5 (H1–H5) have the same fold, including the long loop at the end of H2 (red and green asterisk). Right- super-positioned structure shown with DNA and 8-oxoguanine bound in the active site (green flipped base). Red- NLRP3 1–81 and Rainbow- hOGG1 248-346. **d** Left- NLRP3 (pink) aligned with hOGG1. NLRP3 Lys2 is not shown in the 3D structure, which starts at Met3. Middle- 3D superposed hogg1 with residues important in binding ox-DNA shown. Right- Superposition of nlrp3 and hogg1 domains shown.

Pairwise alignment of NLRP3 with human glycosylase (hOGG1) also showed several residues that were the same or very similar (Fig. 4a). Residues that were the same and important for binding 8-oxoguanine containing DNA were Lys2, Asp21, and Phe75 (NLRP3) and Lys249, Asp268, and Phe319 (hOGG1). Lys249 is mutated to glutamine for the hOGG1 structure shown. The K249Q mutation allows the glycosylase to bind 8-oxoguanine DNA but lacks catalytic activity^17^. The span of residues aligned with hOGG1 corresponds to NLRP3 residues 1–81 which maps to the NLRP3 pyrin domain. The residues from the pairwise alignment were then mapped to the structure using MatchMaker in ChimeraX^18^. The HhH-GPD domain of human glycosylase^19^ shares a fold with the NLRP3 pyrin domain, as evidenced by the alignment of the 3D structures and helix topology of helices 1–5 (Fig. 4c). The residues important in binding and catalysis of ox-DNA for hOGG1^12,20^ were on the same corresponding vicinity for NLRP3 (Fig. 4d). The Lys2 for NLRP3 that corresponds to the Lys249 in hOGG1 is not shown in the NLRP3 3D structure which begins at Met3 (Fig. 4d). Conformational differences between the two structures illustrate a rearrangement of H1 and the long loop immediately after H2 (Fig. 4c, Supplementary Fig. 4, and Supplementary Movies 1, 2).

There are several residues in the NLRP3 pyrin that are mutated in NLRP3-associated autoinflammatory diseases (NLRP3-AIDs) that have the same amino acids in the human glycosylase sequence (Fig. 4b). For example, NLRP3 D21H^21–23^ which is an NLRP3-AID corresponds to human glycosylase Asp268. NLRP3 Muckle-Wells Syndrome mutants D31V^21,24,25^ and V72M^21,24^ correspond to human glycosylase Asp278 and Val317, respectively. The NLRP3 A77V^21^ is an undefined CAPS mutant which maps to human glycosylase A322.

### NLRP3 pyrin domain binds oxidized DNA

**Fig. 5:**
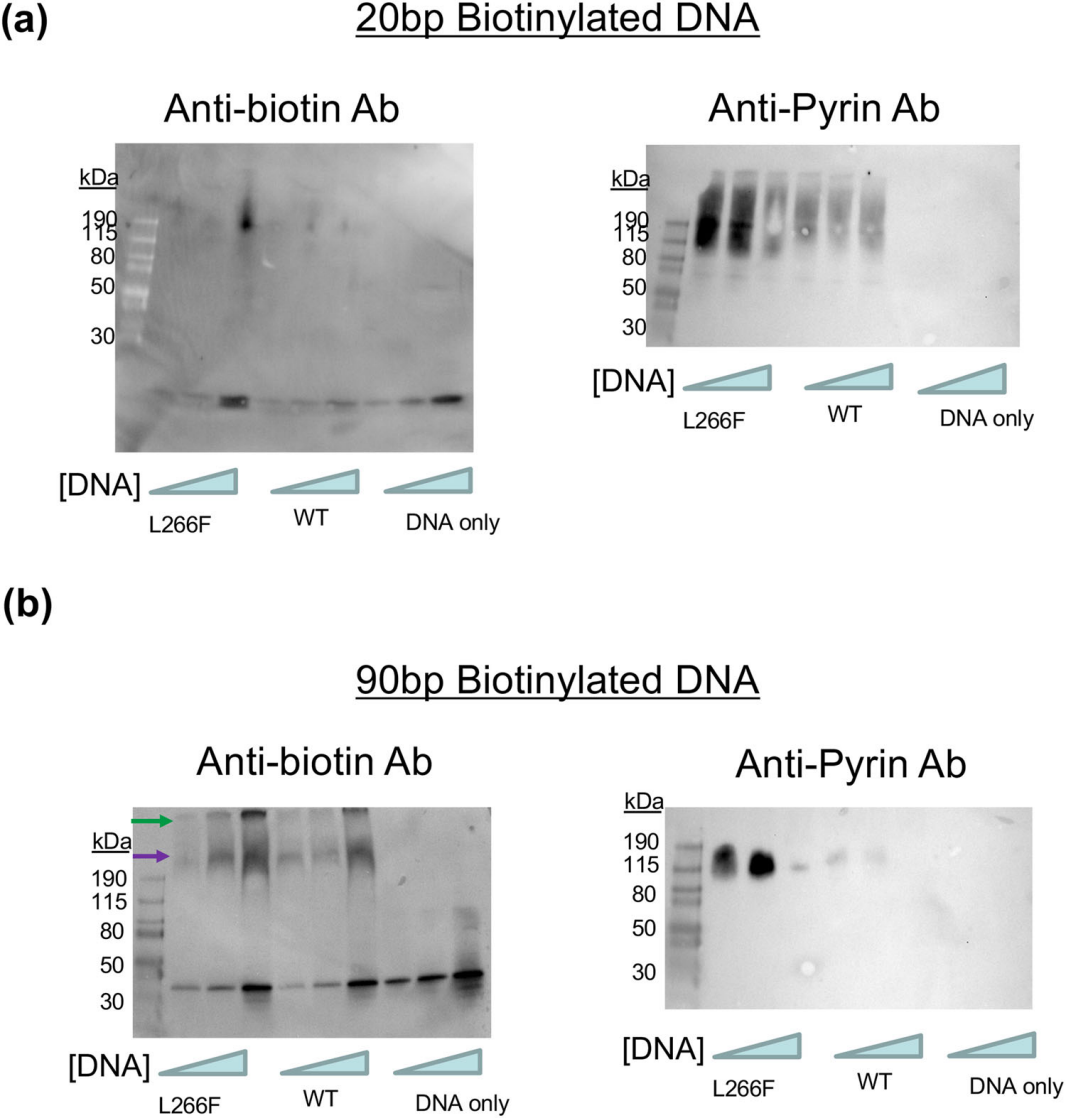
Oxidized mtDNA binds to the pyrin domain of NLRP3. **a** EMSA for ox-DNA 20 bp. Anti-biotin DNA shift or anti-NLRP3 for FCAS mutant, WT, or DNA alone. **b** EMSA for ox-DNA 90 bp. Anti-biotin DNA shift or anti-NLRP3 for FCAS mutant, WT, or DNA alone.

To determine if the NLRP3 pyrin domain could bind ox-DNA, we performed an electromobility shift assay (EMSA) with purified full-length NLRP3 incubated with 20 and 90 bp biotinylated D-loop mtDNA. We observed an upward shift of the DNA mixed with protein compared to DNA alone, indicating DNA was bound to the protein (Fig. 5). The membrane was then stripped to remove the streptavidin antibody and reprobed with a monoclonal antibody that targets the NLRP3 pyrin domain residues 1–93^26^. We could only detect NLRP3 where no shift was observed (Fig. 5). Interestingly, the NLRP3 monoclonal antibody was not able to bind to the complex seen on the anti-DNA image. In the case of both 20 and 90 bp ox-DNA, the monoclonal antibody targeting the pyrin domain only detected NLRP3 in the area immediately surrounding the DNA shift (Fig. 5). Native-PAGE analysis of NLRP3 premixed with oxidized or non-ox-DNA has faint intensity compared to NLRP3 protein alone (Supplementary Fig. 5). This illustrates there is a significant difference in the pyrin domain upon binding such that the monoclonal antibody can no longer recognize pyrin bound to DNA. Free DNA was not visible at the bottom of the anti-NLRP3 membrane indicating the shadow was not caused by the failure to strip the anti-biotin antibody from the membrane. We were not able to detect a shift when NLRP3 was preincubated with a monoclonal antibody against the pyrin domain before adding DNA (Supplementary Fig. 6).

**Fig. 6:**
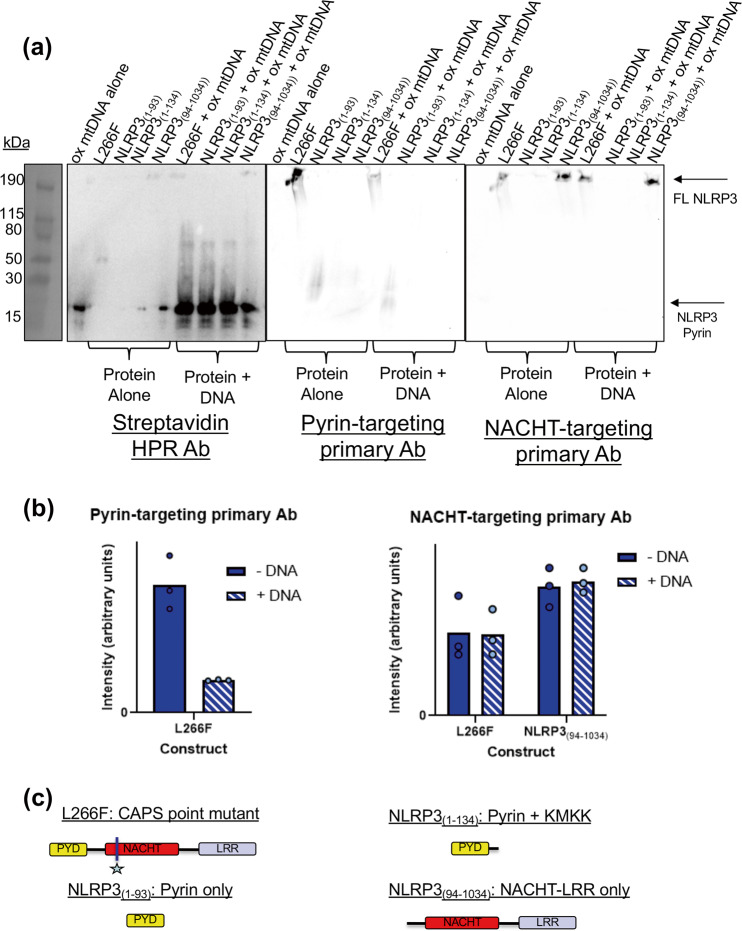
NLRP3-DNA complex band intensity lost with anti-pyrin is recovered using a NACHT-targeting antibody. An EMSA was done using 90-bp biotinylated ox-mtDNA and four different NLRP3 constructs, L266F, NLRP3(1–93), NLRP3(1–134), and NLRP3(94–1034). **a** The same membrane is probed with an antibody targeting the biotinylated ox-DNA (left), an antibody targeting the pyrin domain (middle), and an antibody targeting the NACHT domain (right). **b** Graphs of the intensity of the bands from each respective membrane probe. In the NACHT-targeting graph, bound and free band intensities are relatively the same for the L266F and NLRP3(94–1034) constructs. In the pyrin-targeting graph, there is a roughly 80% decrease in the band intensity comparing the free L266F to the DNA-bound L266F. **c** Visual key for constructs used in this experiment.

These results support that in native conditions used in the EMSA, only NLRP3 unbound oxidized mtDNA can be observed because the pyrin-targeting antibody is prevented from accessing the pyrin domain. To examine if the monoclonal pyrin antibody was indeed being blocked by bound oxidized mtDNA, we repeated the EMSA and reprobed it with a monoclonal antibody against the NACHT domain. After incubating L266F in the presence or absence of ox-DNA, we found the initial loss of NLRP3 band intensity could be rescued using an anti-NACHT antibody (Fig. 6).

To examine the selectivity of the NACHT and pyrin domains for oxidized and non-ox-DNA, we expressed NLRP3_(94-1034)_, which lacks the pyrin domain. Dynabeads were incubated with either biotinylated oxidized or non-oxidized mtDNA to discern if they could pull down purified NLRP3_(94-1034)_. We found NLRP3_(94–1034)_ lacking the pyrin domain could bind both oxidized and non-oxidized mtDNA (Supplementary Fig. 7).

**Fig. 7:**
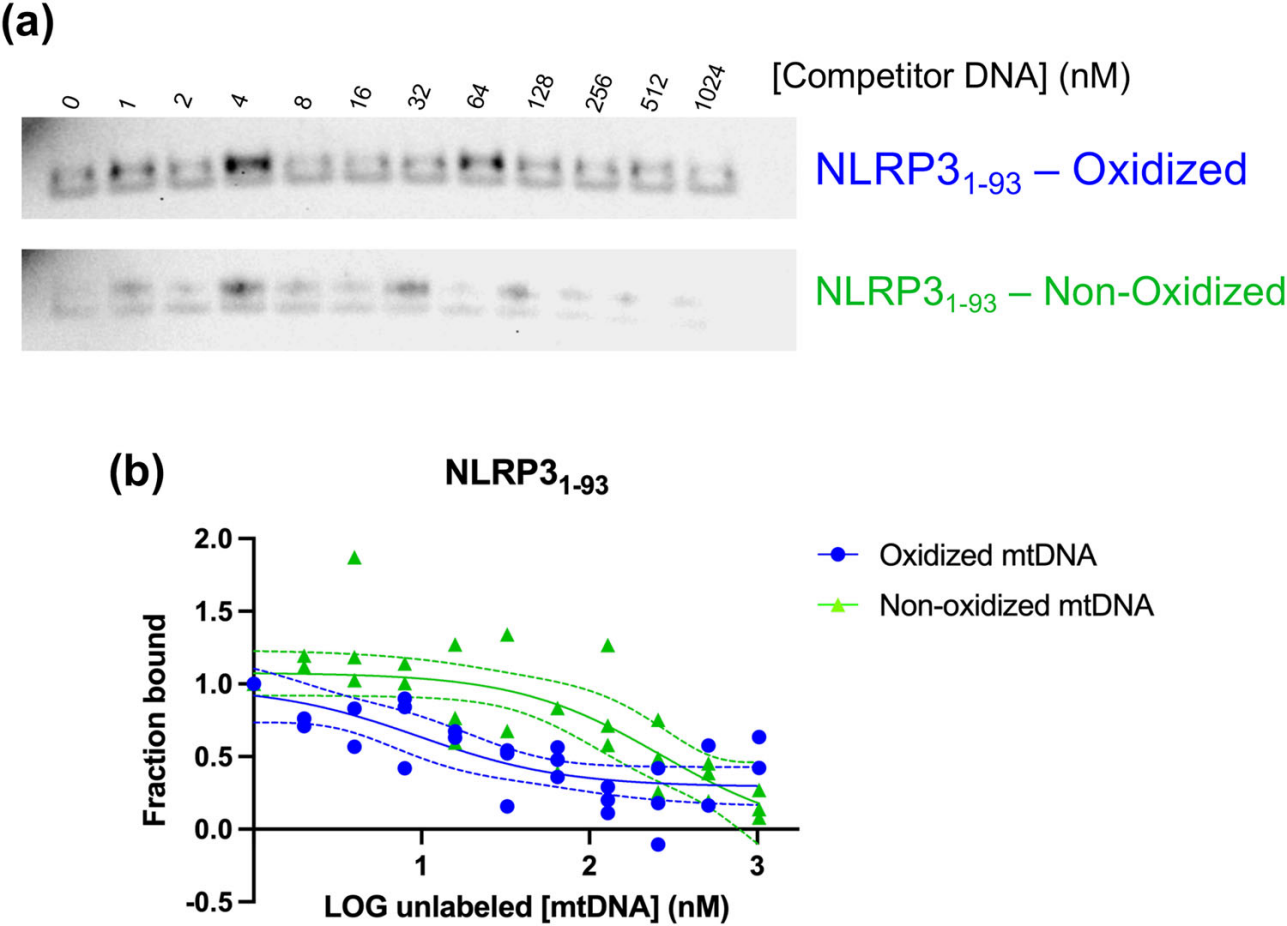
Pyrin domain of NLRP3 prefers oxidized over non-oxidized mtDNA. **a** NLRP31-93 pyrin domain was eluted from biotinylated oxidized or non-oxidized mtDNA coated streptavidin Dynabeads with increasing concentrations of unlabeled oxidized or non-oxidized mtDNA. A representative gel of each membrane of each shown (*n* = 3). **b** Fraction bound vs. LOG unlabeled mtDNA for elution with oxidized or non-oxidized mtDNA. Raw data plotted. The dashed line represents a 95% confidence interval of best-fit line (solid line), *n* = 3.

Expression of NLRP3_(1-93)_, which contains only the pyrin domain, was examined in the competition pulldown assay as originally shown in Fig. 2. NLRP3_(1–93)_ showed strong initial binding to oxidized mtDNA compared to non-oxidized mtDNA, which exhibited faint binding under all conditions tested. The results show NLRP3 pyrin domain has a preference for oxidized mtDNA (Fig. 7). We also note the pyrin domain in the mtDNA competition assay yields two bands at the expected size for the pyrin domain (Fig. 7).

## Discussion

Although many factors have been shown to activate the NLRP3 inflammasome, a bona fide directly interacting ligand has yet to be found. To our knowledge, we report the first study to demonstrate a direct interaction between NLRP3 and an agent known to activate NLRP3 inflammasome and map the binding to the pyrin domain using a monoclonal antibody. It was previously shown that transfected ox-DNA could activate the NLRP3 but not the AIM2 inflammasome^10^. Other studies showed that ox-DNA co-immunoprecipitates with NLRP3 in lysate^11^, but it remained possible that the ox-DNA was attached to some other binding partner and not directly to NLRP3. In cell lysate that contains many other proteins, a DNA shift in the region of NLRP3 suggests that NLRP3 can bind DNA in a competitive environment containing many other proteins (Fig. 1). We have shown that NLRP3 can bind ox-DNA and that the complex is sensitive to stringency which elutes with 1 M NaCl (Fig. 1). We suggest the absence of a detectable DNA shift for L355P and L266F (Fig. 1b, c) is due to differential transfer to the membrane since we can detect binding with other methods (Figs. 2 and 6). Both wild-type NLRP3 and NOMID mutant respond differently when oxidized guanine is incorporated into the DNA. Although wild-type NLRP3 can bind both oxidized and non-ox-DNA, its affinity is higher for non-ox-DNA. On the contrary, NOMID NLRP3 has a higher affinity for ox-DNA (Fig. 2b). This might explain why NOMID is more sensitive to external activators than normal NLRP3.

ROS species exposure to newly synthesized mtDNA^27,28^ promotes NLRP3 inflammasome assembly and activation^10,11,29^. The DNA exits the mitochondria via mitochondrial permeability transition pore (mPTP) and voltage-dependent anion channels (VDAC) and subsequently activates NLRP3^30^. When the mPTP complex is stimulated with ROS, the mitochondria swell, and mitochondrial DNA fragments are released^31^. Although mitochondrial ROS are sufficient for activation, they are not strictly necessary^30,32^. Macrophages treat their mitochondria as expendable and initiate selective mitophagy in the presence of low levels of ROS. However, high levels of ROS cause non-specific mitophagy^33^ enabling ROS to somehow enable inflammasome activation. As DNA in the presence of ROS can lead to oxidized guanine on the DNA, the ox-DNA would be able to interact with NLRP3 subunits. We show NLRP3 can bind both 20 and 90 bp ox-DNA (Fig. 5). Interestingly, the 90 bp sequence has a super-shift that is much higher than 20 bp. It is possible variable stoichiometric units are binding to the much longer 90 bp sequence or that the 90 bp sequence is wrapping NLRP3 and participating in its oligomerization.

There are other documentations for pyrin domain sensing role in the innate immune system. For example, the pyrin domain uses phosphorylation by RhoGTPases to sense virulence factors^34^. Moreover, this would not be the first example of proteins interacting with ox-DNA, as glycosylases can bind both 8-oxodG containing DNA^12^ and also the nucleoside 8-oxodG^35^. We show the pyrin domain can bind oxidized mtDNA (Fig. 7) and that the NACHT domain is also poised for interaction with 8-oxoguanine DNA (Supplementary Fig. 7) with its positive surface and homology to CacOgg (Fig. 3). The exact role ox-DNA plays in NLRP3 inflammasome activation is yet to be determined. We speculate the positive density may position the pyrin-bound oxidized mtDNA for interacting with the ASC pyrin domain. The positive surface might also serve as an allosteric site, given this is near the region of the NACHT domain where the MCC950 inhibitor binds^4^. The interaction may promote the removal of inhibitory interactions between NLRP3 NACHT and LRR domains. Since human glycosylase and NLRP3 share the same fold, we propose NLRP3 Lys2 may serve to excise 8-oxoguanine from mtDNA, which is the role of corresponding Lys249 in hOGG1^20^, which may pave the way for new and innovative NLRP3 inhibitors. The N-terminal region of NLRP3-containing Lys2 fits the canonical definition of an IDR (Supplementary Fig. 3) and has the propensity to osculate between order and disorder. It is possible that this region may become ordered when bound to DNA. Future experiments will probe these molecular determinants of recognition of NLRP3 for mtDNA and 8-oxodGTP using additional site-directed mutagenesis of the DNA binding site, cell-based assays, and cryo-electron microscopy^36^.

## Methods

### NLRP3 expression and purification

Both wild-type NLRP3 and CAPS mutants were cloned in the mammalian expression vector pcDNA3.1HisB. Plasmid was purified using Qiagen Giga Prep up to 2000 ng/μl and subsequently transfected into Expi293 with Expifectamine per manufacturer instructions. Expression was allowed to proceed for 3 days before harvesting. Cells were centrifuged at 1200 rpm. Dead cells were aspirated, and live cells were resuspended in lysis buffer containing (50 mM Tris-HCl pH 7.4, 1 mM PMSF, 1X protease and phosphate inhibitor, 1% EDTA, 300 mM NaCl, 0.1% SDS, 10% glycerol, and 1% Triton X-100). Both protease and phosphatase inhibitors (Roche) were added. Cells were sonicated (2 s on, 8 s off. 40% power, 42 s) and centrifuged at 100,000 × *g*. Lysates were passed through a 0.22 μm filter and then loaded onto a cobalt column. The cobalt column was equilibrated with Buffer A (20 mM Tris-HCl, 200 mM NaCl, 10% glycerol, 1 mM DTT, pH 7.4) and protein was eluted with Buffer B (20 mM Tris-base, 200 mM NaCl, 10% glycerol, 1 mM DTT, 500 mM imidazole, pH 7.4). His-tag purified protein was loaded onto HiLoad 16/600 Superose 6 size exclusion column equilibrated in 20 mM Tris, 200 mM NaCl, 1 mM DTT, 10% glycerol pH 7.4 (Supplementary Fig. 8). Protein was concentrated near 1 mg/ml for assays.

### NLRP3_(1-93)_ mutagenesis, expression, and purification

Site-directed mutagenesis was performed on WT NLRP3 to introduce a stop codon after amino acid 93. NLRP3 _(1–93)_ was cloned in the mammalian expression vector pcDNA3.1HisB. The plasmid was purified using PureLink HiPure Expi Megaprep, yielding a final concentration of 1780.6 ng/μL. The plasmid was transfected into Expi293 cells grown in 250 mL of expression media. Enhancers were added and harvested on day 4, reaching a final cell density of 8.4 × 10^6^ org/ml (80% viable). Cells were centrifuged at 1200 rpm in a swinging bucket rotor (JS-4.750, Beckman). Following centrifugation, dead cells were aspirated, and the pellet was resuspended in PBS to remove residual media. Half of the pellets were used for purification. The pellets were resuspended in 40 mL of lysis buffer (50 mM Tris-HCl pH 7.4, 1 mM PMSF, 1X protease and phosphate inhibitor (Halt), 300 mM NaCl, 0.1% SDS, 10% glycerol, 1% Triton X-100). Cells were sonicated (2 s on, 8 s off, 40% power, 42 s) and then centrifuged 100,000×*g* (70-Ti, Beckman). Lysate was passed through a 0.45 um filter and loaded onto a HisTrap FF crude 5 mL nickel column on the AKTA Avant. The column was equilibrated with Buffer A (20 mM Tris-HCl, 200 mM NaCl, 10% glycerol, 1 mM DTT, pH 7.4) and protein was eluted with Buffer B (20 mM Tris-HCl, 200 mM NaCl, 0.5% NP-40 (Vajjhaa), 10% glycerol, 1 mM DTT, 500 mM imidazole, pH 7.4). Protein was eluted stepwise with 5, 50, and 100% buffer B. The sample was then loaded on a HiLoad 16/600 Superose 6 size exclusion column equilibrated with 20 mM Tris-HCl, 200 mM NaCl, 0.5% NP-40, 1 mM DTT, 10% glycerol pH 7.4 (Supplementary Fig. 9). Protein concentration was determined via Bradford Assay yielding a final concentration of 1 mg/mL.

### Wild-type NLRP3, L266F, NLRP3_(1–134)_, and NLRP3_(94–1034)_ expression and purification

Site-directed mutagenesis was performed on WT NLRP3 to introduce a stop codon after the KMKK sequence following the pyrin domain in NLRP3 (after amino acid 134). NLRP3_(1-134)_ was cloned in the mammalian expression vector pcDNA3.1HisB. The plasmid was purified using PureLink HiPure Expi Megaprep. Cloning of the NLRP3(94-1034) construct was outsourced to Azenta for cloning and mutagenesis. Once we received their plasmid, it was sequence validated, grown at large scale, and purified using PureLink HiPure Expi Megaprep. All proteins in these studies were expressed using the Expi293^TM^ Expression System (Thermo Fisher). Cells grown were grown in Expi293 expression media until they reached a concentration of $3\times {10}^{6}$ cells per milliliter and sustained viability of $\ge$95% live cells. At that time, 1 µg of expression vector was transfected per every 1 mL of cells. Typically, transfections were done between 200 and 300 mL of cells. Once the cells reached viability of $\le$80% live cells, they were harvested by spinning at 300 rpm for 5 min. The supernatant/dead cells were aspirated from the top, and the pellet was washed with cold PBS. The cells were pelleted again at 1200 rpm for 5 min and lysed with 50 mM Tris pH 7.4, 1 mM PMSF, 1x protease and phosphatase inhibitor, 300 mM NaCl, 0.1% SDS, 10% glycerol, and 1% Triton X-100. The lysate was sonicated for 42 s in intervals of 2 s on, 8 s off, then clarified by spinning at 100,00 × *g* for 60 min. The clarified lysate was passed through a 0.45 µm filter and purified using a HisTrap FF crude 5 mL column. The column was pre-equilibrated with 20 mM Tris, 200 mM NaCl, 10% glycerol, 1 mM DTT, and 25 mM Imidazole at pH 7.4. After the sample was loaded onto the column, it was washed with 10 column volumes (CV) of the wash buffer above. Then, using a four-step gradient, the protein was eluted using 20 mM Tris, 200 mM NaCl, 10% glycerol, 1 mM DTT, 500 mM Imidazole, and 0.5% NP-40 at pH 7.4. Peak fractions were pooled and loaded onto a HiLoad 16/600 Superose 6 pg size exclusion column. The column was run in a buffer containing 20 mM Tris, 200 mM NaCl, 10% glycerol, 1 mM DTT, and 0.5% NP-40 at pH 7.4. Due to A280 interference by the NP-40, no interpretable peaks could be observed in the UV trace, so all fractions were run on total protein SDS page gels and western blots using run on NuPAGE™ 4 to 12%, Bis-Tris 1 mM 15-well mini-gels at 200 V. For the western blot, samples were transferred to PVDF membranes, blocked with 2.5% BSA, and probed with either a NACHT-targeting antibody (Cell Signaling) at 1:2000 dilution or pyrin-targeting antibody (AdipoGen) at a 1:10,000 dilution in 2.5% BSA in TBST. Blots were incubated with an HRP-linked secondary (anti-mouse or anti-rabbit, depending on the primary) and imaged using the iBright 1500 Imaging system. Once peak fractions were identified, they were pooled and concentrated using 100 kDa cut-off spin concentrators at cycles of 2000×*g* for 5 min. The final samples were run again on total protein SDS page gels and western blots as a final quality check, as shown in Supplementary Fig. 8.

### Electromobility shift assay

EMSA was performed using the Lightshift Chemiluminescent EMSA Kit (Thermo Fisher)^37^. D-loop mtDNA with oxidized guanine was synthesized by IDT. The 100 μM stock of Ox-DNA was diluted twice at 1:100 and once at 1:10. NLRP3 protein extract was serially diluted twofold from 0.225 μg/μL to be used in ten reactions. Each 20 μL reaction consisted of NLRP3 protein extract or purified protein, 1 × 10^−5^ μM oxidized mtDNA, and binding buffer (50 mM Tris, 100 mM NaCl, 2 mM MgCl_2_, 2 × 10^−5^ mg/ml sonicated salmon sperm DNA, 12% glycerol, and pH 7.4). Samples were incubated at 4 °C overnight and run with native conditions on 4–20% Tris/glycine gels. Protein-DNA complexes were transferred to the Biodyne B membrane and UV-crosslinked. Membranes were blocked in 5% BSA for 1 h, washed three times for 10 min, and probed for anti-biotin with streptavidin-HRP antibody (BD Pharmingen). After three additional washes, membranes were exposed to an anti-mouse secondary antibody for 1 h, washed three times for 10 min, and subjected to chemiluminescence. The intensity of the HRP reaction was captured on an x-ray film (KODAK) and developed. Subsequently, membranes were stripped with 25 mM glycine, 1% SDS pH 2.4, and reprobed for human NLRP3 pyrin domain (Adipogen)^2^.

### Pulldown and elution assay

Protocol for pulldown and elution was adopted from a previous study^38^. Briefly, Dynabeads M-280 Streptavidin (Thermo Fisher) were incubated with 200 ng/μL biotinylated mtDNA (oxidized or non-oxidized) in binding buffer (50 mM Tris, 100 mM NaCl, 2 mM MgCl_2_, 12% glycerol, and pH 7.4) overnight at 4 °C. After overnight incubation, beads were washed 5 times to remove unbound DNA. Subsequently, 200 μl NLRP3 protein was incubated with DNA-coated beads overnight. To remove unbound protein, the sample was washed four to five times until the protein concentration was undetected in the supernatant and verified by gel electrophoresis. For elution with salt, beads were placed in increasing concentrations of NaCl with eluted (unbound) fraction analyzed by SDS gel electrophoresis. With respect to DNA elution experiments, the solution containing beads, DNA, and protein was divided into equal volumes for each elution concentration. Protein was eluted from the beads following incubation with non-biotinylated (unlabeled) mtDNA at 4 °C overnight. The bound fraction was analyzed by placing beads in NuPAGE LDS buffer with 1X NuPAGE reducing agent at 95 °C for 10 min and analyzed by western blot with a monoclonal antibody for NLRP3 pyrin domain (Adipogen)^2^.

### Multiplexed dynabead pulldown

This method was used in Supplementary Fig. 7 with NLRP3 _(94–1034)_ protein. This more qualitative method is helpful for analyzing many proteins at the same time. For each reaction, 20 μL of Dynabeads™ M-280 Streptavidin slurry was removed and washed with binding buffer (50 mM Tris, 100 mM NaCl, 2 mM MgCl_2_, 12% glycerol, and pH 7.4) using a DynaMag-2 magnet. Beads were batch bound to 200 μL of 1 μM biotinylated non-oxidized or oxidized DNA rotating at 4 °C overnight, which was diluted 1:100 in a binding buffer from a 100 μM stock solution. The next day, beads were separated from any unbound DNA on the DynaMag-2 magnet and subsequently washed 5 times with binding buffer. On the last wash, beads/binding buffer slurry were separated out into a PCR plate in 20 μL aliquots per reaction and separated using a DynaMag™−96 Side magnet. Binding buffer was removed and 0.2 mg/mL of protein in 20 μL was added to each tube with beads. The reaction was incubated at 4 °C overnight. The next day, the beads were separated from any unbound protein on the DynaMag™−96 Side magnet, and the unbound fraction was saved for western blot analysis. The beads were washed 5 times in binding buffer or until a concentration reading read close to 0 mg/mL. The last wash was saved to confirm any protein was undetectable in that wash via western blot. The beads were then suspended in a buffer for the SDS page and subsequent western blotting. The unbound protein, last wash, and a sample of concentration-matched free protein (that did not participate in the reaction) was also set up in the buffer for the SDS page. The samples were run on NuPAGE™ 4 to 12%, Bis-Tris 1 mM 15-well mini-gels at 200 V. Samples were transferred to PVDF membrane, blocked with 2.5% BSA, and probed with either a NACHT-targeting antibody (Cell Signaling) or pyrin-targeting antibody (AdipoGen), both at an either a 1:2000 or 1:10,000 dilution in 2.5% BSA in TBST respectively. Blots were incubated with an HRP-linked secondary (anti-mouse or anti-rabbit, depending on the primary) and imaged using the iBright 1500 Imaging system.

### Restoring NLRP3-mtDNA complex signal in EMSA using a NACHT antibody

For each protein, 18 μL at >1 mg/mL was incubated with 2 μL of 1 μM biotinylated DNA for 1 h at 4 °C. During the reaction, an Invitrogen™ Novex™ WedgeWell™ 4–12%, Tris-Glycine gel was pre-run for 30 min at 225 V. Samples were loaded, and the gel was run for 30 min at 225 V. Samples were transferred to a PVDF membrane, blocked using 2.5% BSA in TBST, and probed with a Streptavidin-HRP antibody (BD BioScience) at a 1:2000 dilution in 2.5% BSA in TBST. The membrane was analyzed using the iBright 1500 Imaging system, then stripped with 25 mM glycine, 1% SDS pH 2.4, and rocking at room temperature for 40 min. The blot was rinsed with deionized H_2_O, and the stripping buffer was removed by washing with TBST for 45 min, changing the buffer every 5 min. The membrane was then blocked with 2.5% BSA and probed with a pyrin-targeting primary antibody (AdipoGen) diluted 1:10,000 in 2.5% BSA in TBST. The membrane was washed with TBST for 5 min three times, incubated with an anti-mouse HRP secondary, and analyzed again on the iBright 1500 Imaging system. Subsequently, the membrane was blocked and reprobed with an anti-NACHT rabbit monoclonal antibody (Cell Signaling) diluted 1:2000 in 2.5% BSA in TBST. The membrane was washed with 1x TBST for 5 min three times, incubated with an anti-rabbit secondary, and then analyzed on the iBright 1500 Imaging system.

### Analysis of intrinsically disordered regions across NLRs

Using the D^2^P^2^ Database of Disordered Protein Predictions, intrinsically disordered regions (IDRs) of NRLP1-14 were analyzed. By searching the UniProtKB database and selecting the BLAST feature, FASTA files of the protein sequences of NLRP1-14 were compiled. Then, one by one, they were inputted into the D^2^P^2 “^Match Amino Acid Sequence” protein search tool. This search returned a matched sequence for each NLR query. The results provided maps incorporating the sequence length, domain locations, post-translational modification sites, predicted disorder agreements, and identification of the type of predicted disorder. These maps were compared to one another to identify where most IDRs were predicted to be, and which NLRs most closely resembled the IDR predictions of NLRP3.

### Sequence alignment and homology modeling

A pairwise alignment between NLRP3 (PDBID 7pzc) and *C. acetobutylicum* 8-oxoguanine DNA glycosylase (PDBID 3F0Z) or human glycosylase (PDBID 1ebm) was performed using EMBOSS Needle^39^. Corresponding amino acids for the glycosylase mapped mostly to NLRP3 NACHT domain (Fig. 3). Pairwise alignment of NLRP3 to human glycosylase (PDBID 1ebm) mapped solely to the NLRP3 pyrin domain (Fig. 4). Corresponding regions of the glycosylase (PDBID 1ebm) were superposed onto NLRP3 using MatchMaker in UCSF ChimeraX^18^. The RMSD between 18 pruned atom pairs vs. across all 71 atom pairs was 1.17 and 11.97 Å, respectively. Multiple sequence alignments were performed with Emboss Needle (Supplementary Fig. 10).

### Statistics and reproducibility

For mitochondrial DNA competition assays, at least three independent biological replicates from separately loaded Dynabeads were used. The 95% confidence of the best-fit line for each variable is graphed to illustrate significance. Individual points are shown in lieu of means with Y-error bars for triplicate data. Representative uncropped gel blots for data used are shown herein (Supplementary Figs. 8, 9,11, 12).

## Supplementary information

McNulty_Peer Review File

Supplementary Material

Description of Additional Supplementary Files

Supplementary Movie 1

Supplementary Movie 2

## Data Availability

Gels analyzed in the study are available uncropped in the supplementary material. All other data that support the findings of this study are available from the corresponding author upon reasonable request.
