# Author Correction: Differential binding of NLRP3 to non-oxidized and Ox-mtDNA mediates NLRP3 Inflammasome Activation

**DOI:** 10.1038/s42003-023-05019-2

**Published:** 2023-06-12

**Authors:** Angela Cabral, Julia Elise Cabral, Angelina Wang, Yiyang Zhang, Hailin Liang, Donya Nikbakht, Leslie Corona, Hal M. Hoffman, Reginald McNulty

**Affiliations:** 1 Department of Molecular Biology and Biochemistry, University of California Irvine, Steinhaus Hall, Irvine, CA 92694-3900 USA; 2 Division of Pediatric Allergy, Immunology, and Rheumatology, Rady Children’s Hospital of San Diego, University of California, San Diego, San Diego, CA USA

**Keywords:** Innate immunity, Structural biology

Correction to: *Communications Biology* 10.1038/s42003-023-04817-y, published online 30 May 2023.

In the original version of this Article, the Abstract mistakenly used the acronym, “NOMIDFCAS” to refer to neonatal-onset multisystem inflammatory disease:

“The NLRP3 Neonatal-Onset Multisystem Inflammatory Disease (NOMIDFCAS) gain of function mutant could bind non-oxidized mtDNA but had higher affinity for Ox-mtDNA compared to WT with an IC50 of 8.1 nM”.

This sentence should instead read as:

“The NLRP3 Neonatal-Onset Multisystem Inflammatory Disease (NOMID) gain of function mutant could bind non-oxidized mtDNA but had higher affinity for Ox-mtDNA compared to WT with an IC50 of 8.1 nM”.

This error has now been corrected in the PDF and HTML versions of the Article.

