# Supplementary material for: Differential Binding of NLRP3 to non-oxidized and Ox-mtDNA mediates NLRP3 Inflammasome Activation
Source: Commun Biol. 2023 May 30;6:578. doi: 10.1038/s42003-023-04817-y (PMC10229695; doi:10.1038/s42003-023-04817-y)

Supplementary Results

NLRP3 oligomer and monomer can both be isolated from the same protein purification

From the data in the total protein SDS page and western blots of the Superose 6 size exclusion column run on wild type NLRP3, we were able to isolate both the oligomerized and monomeric fraction of NLRP3 (Supplementary Figure 8). Fractions A1-12 were analyzed for the oligomerized NLRP3 fraction. Based off the band intensity and purity of each fraction, we decided A2 and A3 had most of the purified oligomer by analysis of the band(s) near 120 kDa, the expected size of our protein (Supplementary Figure 8a). These fractions were pooled and concentrated using a 100 kDa cutoff concentrator to try and remove any lower-order contamination, and the resulting sample was run again on a total protein SDS page and western blot (Supplementary Figure 8a). The sample appeared relatively pure, and highly concentrated. The yield from this purification was ~500 µg of oligomerized NLRP3 protein at a concentration of 1.5mg/mL. The same procedure was done for the monomeric NLRP3 fraction, but fractions C1-C12 were analyzed instead. Based on the gel and western of all these fractions, fraction C6 seemed to have the highest intensity at 120 kDa with the least amount of contamination (Supplementary Figure 8b). After concentration, this protein yielded ~350 µg of monomeric NLRP3 protein at a concentration of 2.1 mg/mL with relatively low contamination by other proteins.

NLRP3$_{(1\text{-}93)}$ Purification and Binding Assay Results

NLRP3$_{(1\text{-}93)}$ was purified with affinity chromatography and size exclusion chromatography (SEC). Following SEC, fractions were analyzed through SDS total protein and western blot, showing purified protein roughly 14 kDa (Supplementary Figure 9). Purified NLRP3$_{(1\text{-}93)}$ was used in the Dynabead pulldown assay with non-oxidized and oxidized mtDNA. The fraction of protein bound to the beads was analyzed in triplicate in both assays with iBright software and compared in PRISM(Figure 7). All data is normalized to the second lane and plotted against the log of the concentration of competitor mtDNA. Analysis shows that NLRP3$_{(1\text{-}93)}$ has a stronger initial binding of oxidized mtDNA than non-oxidized mtDNA. This indicates the pyrin domain's preference for oxidized mtDNA. Representative full membranes for both assays are included (Supplementary Figure 12).

NLRP3$_{(94\text{-}1034)}$ can bind both oxidized and non-oxidized mtDNA

To analyze how an NLRP3 construct lacking a pyrin domain interacted with DNA, the protein was incubated with either oxidized or non-oxidized biotinylated mtDNA bound to streptavidin beads. After a series of washes, the bound fraction was analyzed using a NACHT-targeting primary antibody on a western blot (Supplementary Figure 7). We saw that, compared to a concertation matched free protein control lane, no protein was detected in the pullback (or unbound) fraction, suggesting all protein added to the DNA/beads was bound. There was also no protein detected in the last wash, suggesting that any protein we do see in the bound fraction lane is bound to the DNA/beads, and not in the binding buffer or solution around the

beads. Finally, the bound fraction was also analyzed. For the non-oxidized DNA, a band of similar intensity to the free protein band was seen in the bound fraction lane (Figure S7a). For the oxidized DNA, a band of similar intensity to the free protein band was seen in the bound fraction lane (Supplementary Figure 7b). These results suggest that an NLRP3 construct lacking a pyrin domain can bind both oxidized and non-oxidized mtDNA.

Loss of NLRP3-mtDNA complex signal can be restored by using a NACHT-targeting antibody

Figure 5 suggests the loss in band intensity of NLRP3-mtDNA complex samples in EMSA experiments is because the pyrin-targeting primary antibody is blocked by the interaction between NLRP3 and mtDNA. To further prove this, we wanted to see if we could rescue this signal by probing with a primary antibody for the NACHT domain. Four different constructs (L66F, $NLRP3_{(1-93)}$ $NLRP3_{(1-134)}$, and $NLRP3_{(94-1034)}$ (Figure 6C)) were all incubated with biotinylated oxidized DNA (biot-ox mtDNA) and run under native conditions on a Tris/Glycine gel. Samples were transferred onto a PVDF membrane for western blot analysis (Figure 6). Initially, the membrane was probed with a streptavidin-HPR antibody to observe any DNA shift. A shift is observed in both the L266F and $NLRP3_{(94-1034)}$ samples incubated with biot-ox mtDNA Figure 6a). When the membrane was stripped and probed with an antibody targeting the pyrin domain, we see a loss in intensity with both the L266F and $NLRP3_{(1-93)}$ samples when bound to biot-ox mtDNA (Figure 6b). We also see no bands for the $NLRP3_{(94-1034)}$ construct, supporting the specificity of this antibody for the pyrin domain. The same membrane was stripped again, and this time probed with an antibody targeting the NACHT domain. The intensity of the L266F-DNA and $NLRP3_{(94-1034)}$ complex bands are now comparable to those of their protein alone counterparts (Figure 6b). This data supports our hypothesis that pyrin-targeting antibody binding is blocked by the NLRP3-DNA complex and can be rescued by using an antibody targeting the NACHT domain.

NLRP6, 10, and 12 show similar regions of disorder to NLRP3

Using the D^2P^2 database, we analyzed the predicted regions of disorder across NLRP1-14. After examining all the predictions, and comparing them to NLRP3, the disordered regions that map most similarly to NLRP3 are present in NLRP6, NLRP10, and NLRP12 (Supplementary Figure 3). In all three (and in NLRP3) most of the intrinsically disordered region is clustered in the linker region between the pyrin and the NACHT domains. Interestingly, these are the same NLRs that are the closest to NLRP3 sequentially, as evident by the phylogenetic tree in Supplementary Figure 10C.

NLRP3 Alignment Results

Several amino acids involved in binding oxidized DNA are identical in NLRP3 and hOGG1 (Supplementary Figure 10a). Multiple sequence alignment of NLR pyrin domains, residues 1-100, indicate that these residues are not all conserved in any other family member. Thus, these amino acids are unique to NLRP3 (Supplementary Figure 10b). The phylogenetic tree of full length NLR family members shows that NLRP6, 10, and 12 are the most similar to NLRP3

(Supplementary Figure 10C). These NLR's are also the most similar to NLRP3 in their IDR within the linker region between the pyrin and NACHT domain.

Supplementary Figure 1: NLRP3 is not expressed in Expi293 cells.

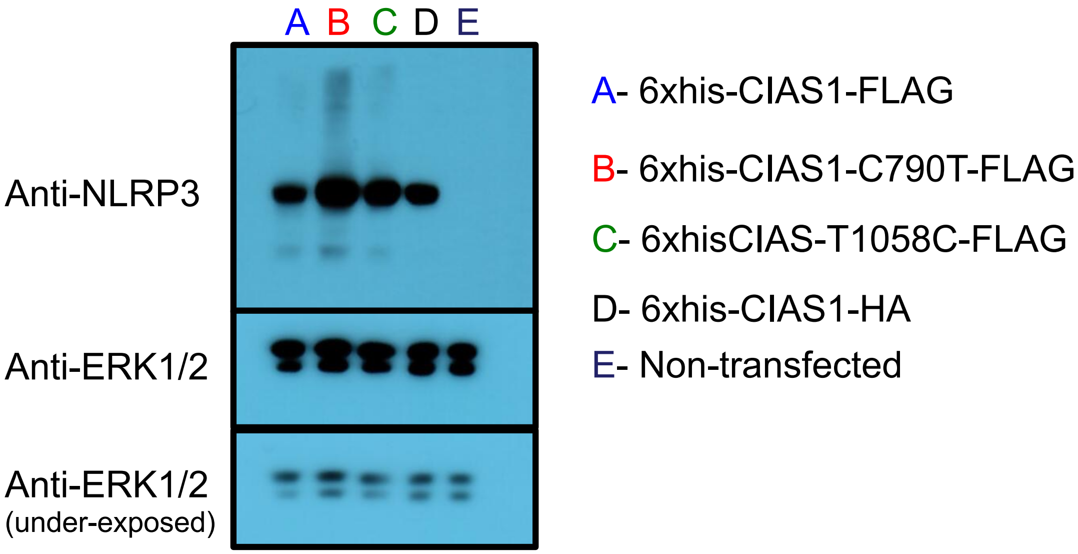

NLRP3 expression detected in (a) WT,(b) NOMID – C790T, and (c) FCAS – T1058C mutants. E- Expi293 cells do not have endogenous NLRP3. Constructs are named as originally provided from the Hofmann lab. Cold-induced autoinflammatory syndrome 1 (CIAS1) is synonymous with NLRP3

Supplementary Figure2 : oxDNA can interact with proteins in Expi293 cell extract not containing NLRP3

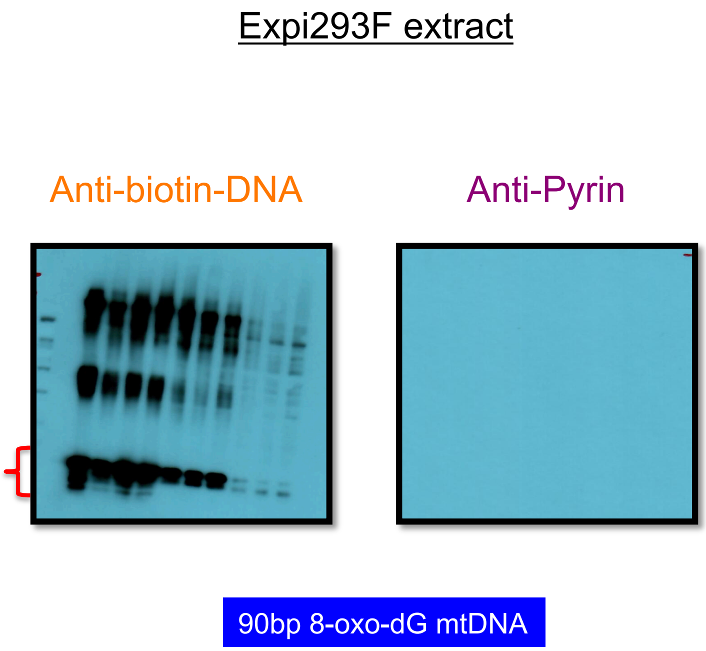

A western blot of Expi293F cells incubated with 90bp oxDNA and with an anti-biotin antibody (left), then re-probed with an anti-pyrin NLRP3 antibody (right). Free oxidized mtDNA (red bracket) shifts to interact with other proteins in Expi293F extract, but there is no NLRP3 present in the extract (right).

Supplementary Figure 3: Most of NLR intrinsic disorder regions (IDR's) are between pyrin and NACHT domains

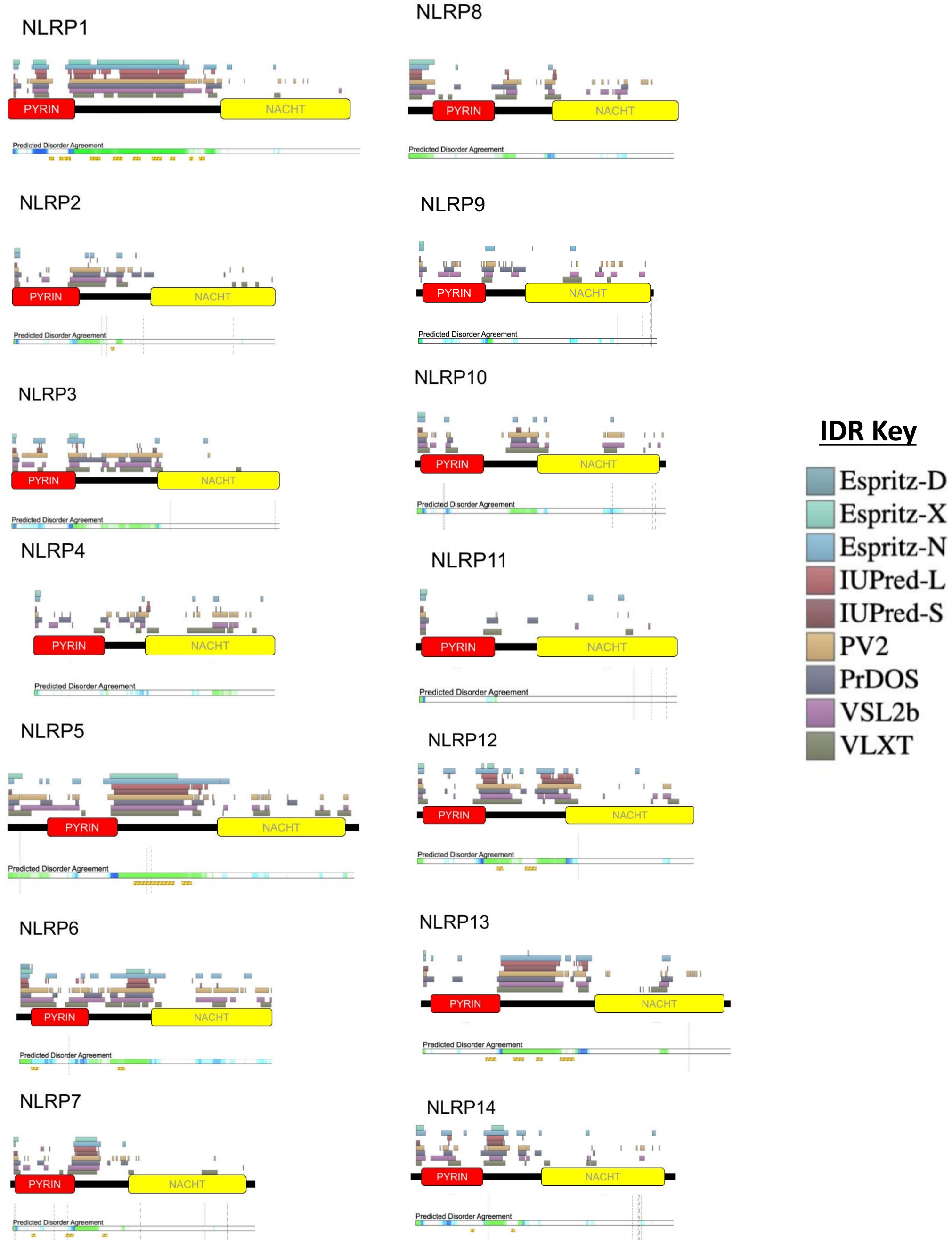

Intrinsically disordered regions of NLRP1-NLRP14 from the beginning of the sequence through the pyrin (red) and NACHT (yellow) domains. Results from several disorder predictor programs are superposed above, color-coded with the key on right. IDR's of NLRP6, 10, and 12 are most consistent with NLRP3 in the distal half of the linker region

Supplementary Figure 4: Conformation change from NLRP3 to hOGG1

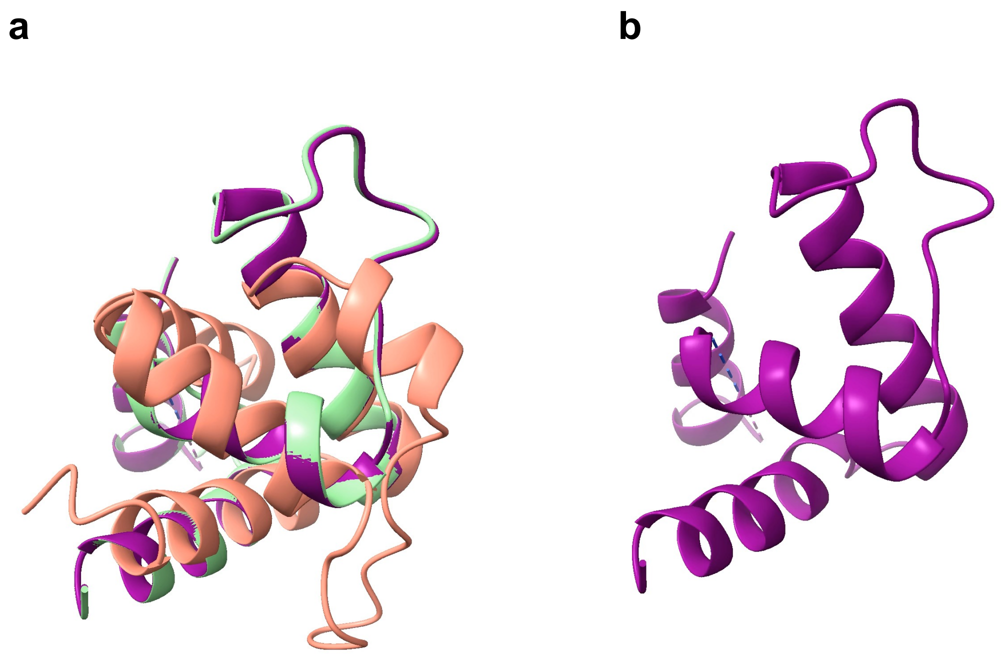

NLRP3 pyrin domain (pink) and hOGG1 (green). (a) Superposition and morph movie (purple) and (b) morph movie alone.

Supplementary Figure 5: Pyrin domain-specific NLRP3 antibody blocked from binding mtDNA

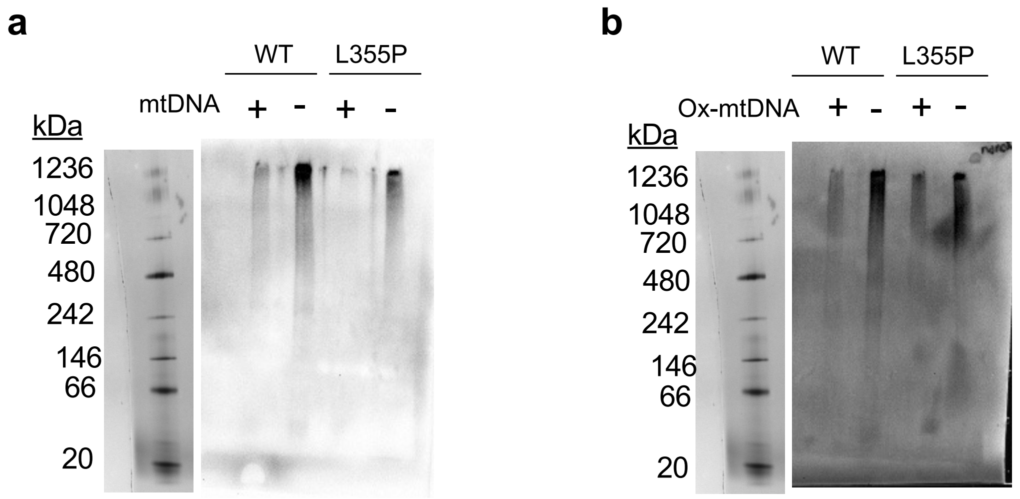

Native-PAGE western blot for both WT and L355 with native ladder. Both proteins were run alone or with non-oxidized mtDNA (a) or oxidized (Ox) mtDNA (b).

Supplementary Figure 6: Protein bound to anti-pyrin antibody domain does not shift in EMSA

a

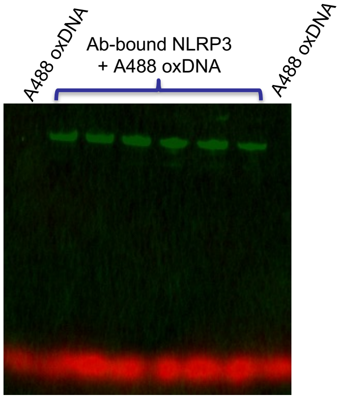

b

<u>Oxidized DNA 90bp</u>

AATCTACCATCCTCCGTGAAACCAACAACCCGCCCACCAATGCCCC

TCTTCTCGCTCCGGGCCCATTAAACTTGGGGGTAGCTAAACTGA

<u>Oxidized DNA 20bp</u>

AATCTACCATCCTCCGTGAA

(a) To support that oxDNA and the antibody against the pyrin domain compete for the same binding region on NLRP3, NLRP3 was preincubated with pyrin-targeting primary Ab before incubating with Alexa 488 labeled oxDNA (red). The complex was then probed with an Alexa 700 labeled secondary (green). The oxDNA alone runs near the bottom of the gel, and the NLRP3 runs near the top of the gel. The middle of the gel shows replicates of the NLRP3-Ab incubated with A488 oxDNA, where no notable shift was observed for the oxDNA. (b) Sequences of the 90bp (top) and 20bp (bottom) ox-DNA used in these studies. Red G’s denote the location of 8-oxo-dGTP.

Supplementary Figure 7: NLRP394-1034 binds both oxidized and non-oxidized mtDNA

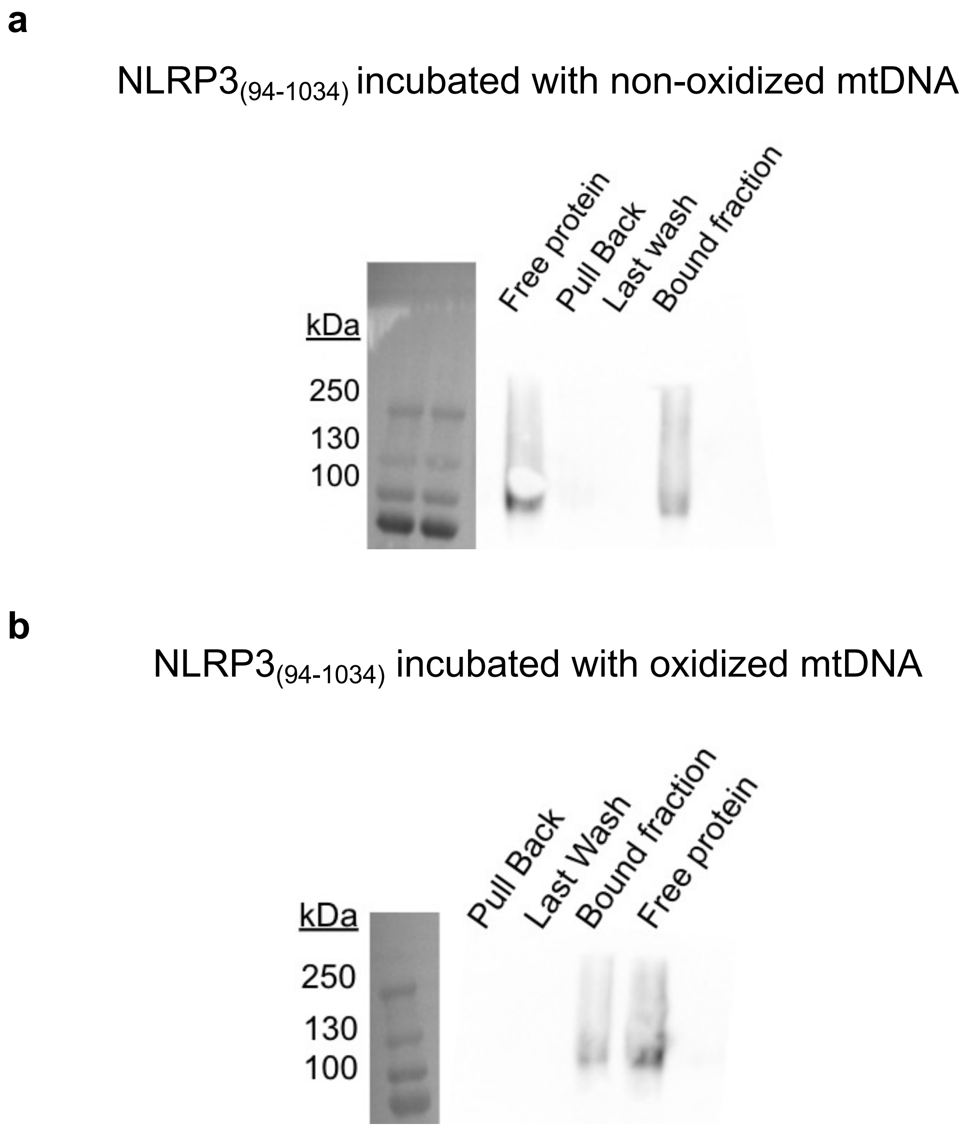

Biotinylated oxidized (a) and non-oxidized (b) mtDNA used to pull down protein pulldown NLRP3$_{(94\text{-}1034)}$ using streptavidin Dynabeads. The bound fraction was analyzed via western blot using an antibody against the NACHT domain.

Supplementary Figure 8: Purification of wild-type full-length NLRP3 using Superose 6 SEC

a

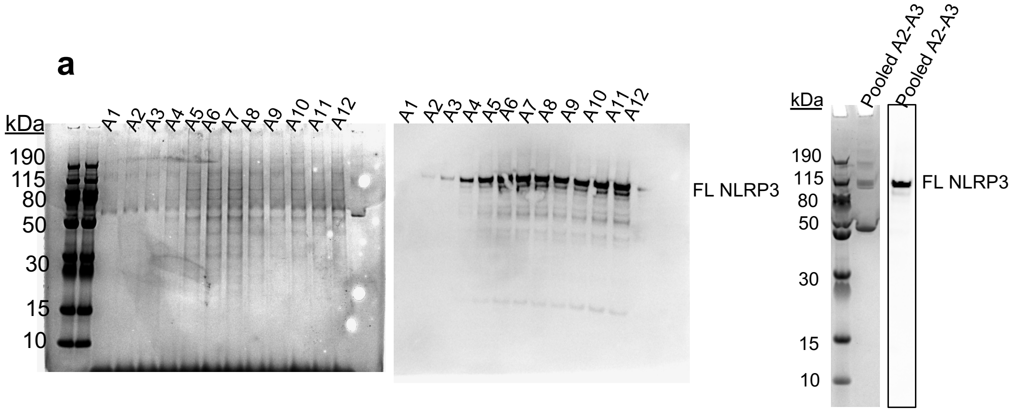

b

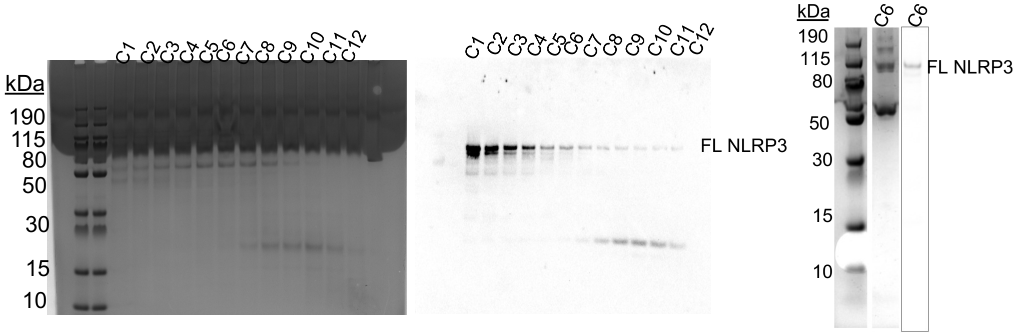

(a) Purification of oligomerized NLRP3. Initial size exclusion “A” fractions were analyzed: SDS Coomassie gel of all “A” fractions; middle- western blot of all “A” fractions; right- SDS Coomassie gel and western blot of final pooled and concentrated sample. (b) Purification of monomeric NLRP3. Initial size exclusion “C” fractions were analyzed: Left- SDS Coomassie gel of all “C” fractions; middle- western blot of all “C” fractions; right- SDS Coomassie gel and western blot of final concentrated sample.

Supplementary Figure 9: NLRP3$_{1-93}$ size exclusion chromatography (SEC) purification

a

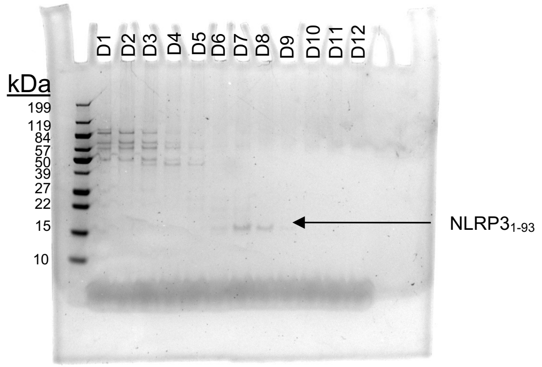

b

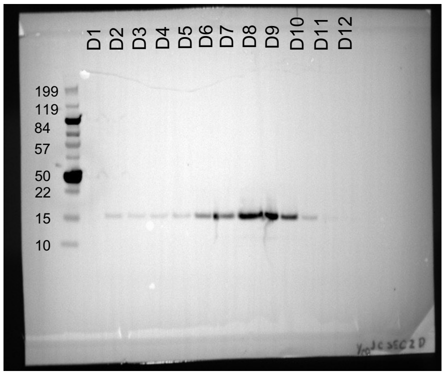

Following affinity chromatography, SEC was performed with 16/600 Superose 6 column. (a) 4-12% BisTris SDS total protein gel. Expected roughly 14 kDa. (b) Western on fractions D1-D12 off SEC column visualized on PVDF membrane.

Supplementary Figure 10: Multiple Sequence Alignment of NLR Family Proteins

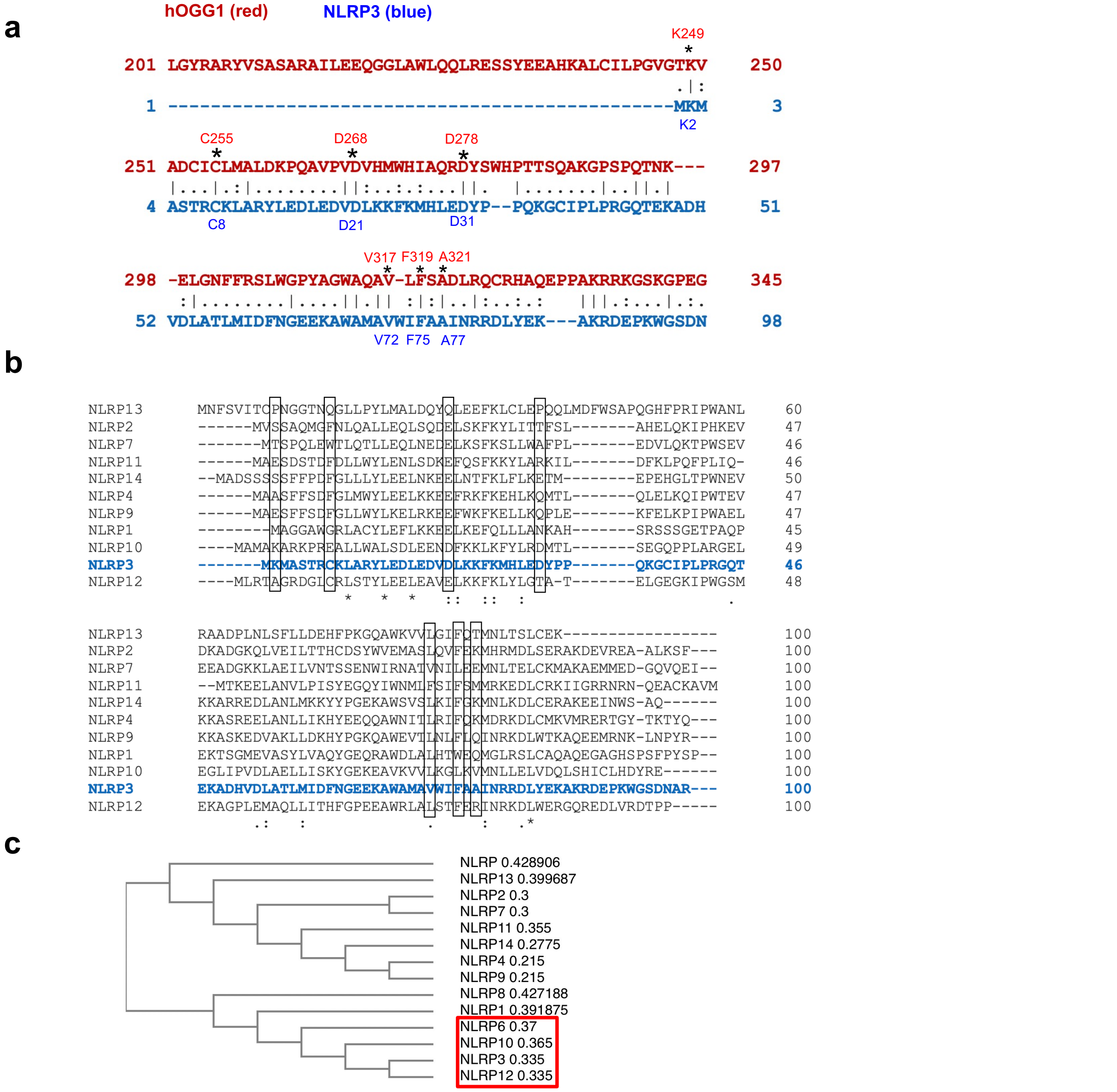

(a) Comparison of NLRP3 pyrin domain and hOGG1. Conserved residues involved in DNA binding are indicated by asterisks. (b) Alignment of pyrin domains (amino acids ranging 1-100) in NLR family 1-14. NLRP 5, 6, and 8 are excluded because they do not map to the pyrin domain region of the other aligned proteins. Black boxes indicate conserved residues involved in DNA binding between all NLRs which are also present in hOGG1. (c) Phylogenetic tree from alignment of full length NLRP1-14. NLRP3, 6, 10, and 12, which map similarly in their IDR regions (Supplementary Figure 3), are also more related in sequence (red box).

Supplementary Figure 11: Uncropped figures from Figure 1 B and C

Anti-Pyrin

T1058C (L355P)

a

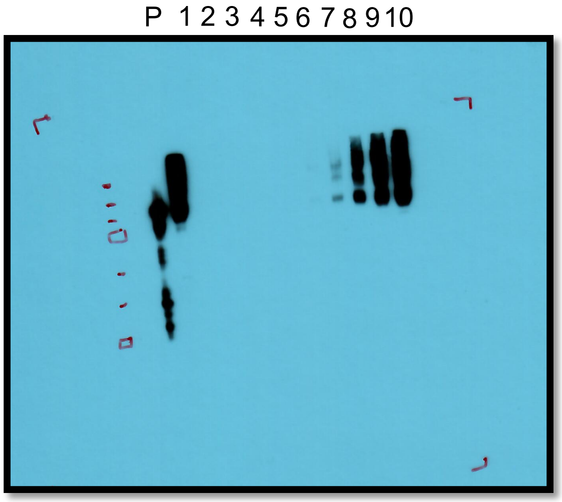

Anti-Pyrin

C790T (L266F)

b

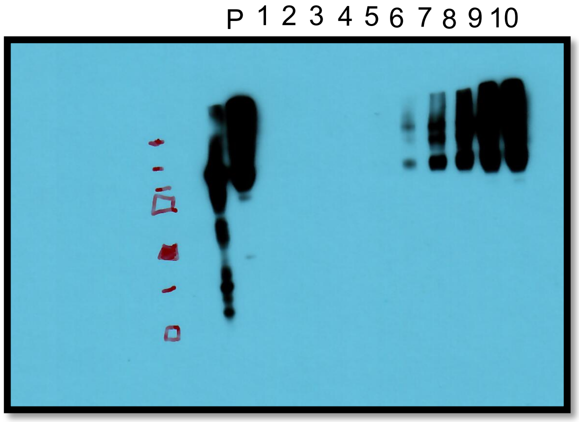

Supplementary Figure 12: Uncropped western blot of Fig. 7

NLRP3$_{1-93}$ Oxidized

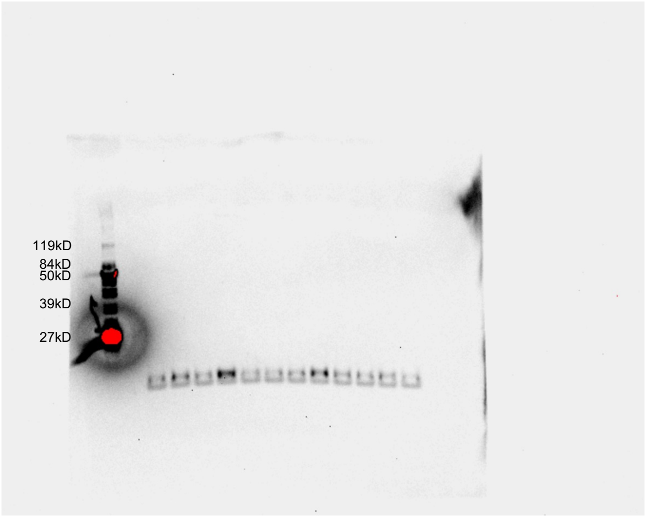

NLRP3$_{1-93}$ – Non-Oxidized

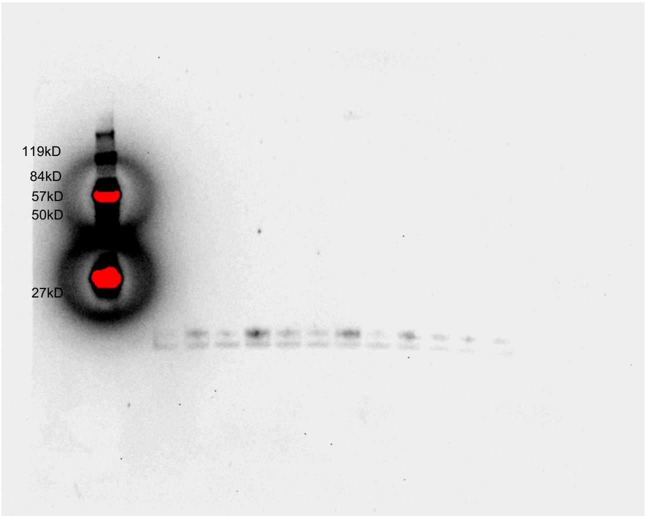

Supplement: Supplementary file 2 — Supplementary Material [file 42003_2023_4817_MOESM2_ESM.pdf]
