# Supplementary material for: Differential Binding of NLRP3 to non-oxidized and Ox-mtDNA mediates NLRP3 Inflammasome Activation
Source: Commun Biol. 2023 May 30;6:578. doi: 10.1038/s42003-023-04817-y (PMC10229695; doi:10.1038/s42003-023-04817-y)
Supplement: Supplementary file 3 — Description of Additional Supplementary Files [file 42003_2023_4817_MOESM3_ESM.pdf]

Description of Additional Supplementary Files

File name: Supplemental Figure Movie 1

Description: Morph movie from NLRP3 pyrin to hOgg1 with starting and ending files.

File name: Supplemental Figure Movie 2

Description: Morph movie from NLRP3 pyrin to hOgg1.
