# Supplementary material for: Differential Binding of NLRP3 to non-oxidized and Ox-mtDNA mediates NLRP3 Inflammasome Activation
Source: Commun Biol. 2023 May 30;6:578. doi: 10.1038/s42003-023-04817-y (PMC10229695; doi:10.1038/s42003-023-04817-y)
Supplement: Supplementary file 1 — McNulty_Peer Review File [file 42003_2023_4817_MOESM1_ESM.pdf]

nature portfolio

Peer Review File

Differential Binding of NLRP3 to non-oxidized and Ox-mtDNA mediates NLRP3 Inflammasome Activation

Open Access This file is licensed under a Creative Commons Attribution 4.0 International License, which permits use, sharing, adaptation, distribution and reproduction in any medium or format, as long as you give appropriate credit to the original author(s) and the source, provide a link to the Creative Commons license, and indicate if changes were made. In the cases where the authors are anonymous, such as is the case for the reports of anonymous peer reviewers, author attribution should be to 'Anonymous Referee' followed by a clear attribution to the source work. The images or other third party material in this file are included in the article's Creative Commons license, unless indicated otherwise in a credit line to the material. If material is not included in the article's Creative Commons license and your intended use is not permitted by statutory regulation or exceeds the permitted use, you will need to obtain permission directly from the copyright holder. To view a copy of this license, visit http://creativecommons.org/licenses/by/4.0/.

Reviewers' comments:

Reviewer #1 (Remarks to the Author):

Wang and colleagues present a study focusing on the question of whether NLRP3 is able to directly bind oxidised or non-oxidised DNA. Oxidised mitochondrial DNA (mtDNA) has been previously proposed to be a general ligand/binding partner required for activation of the NLRP3 inflammasome, but whether NLRP3 can directly bind oxDNA or DNA was not determined, and that question is addressed in this study. Whilst the authors provide data suggesting that NLRP3 does indeed directly interact with oxDNA, further experiments and controls are still required to conclusively demonstrate their hypothesis. We would also comment that the authors conclusions from their data are, in general, slightly strong for the evidence provided, and so should be rephrased to include alternative hypothesises, or reduced in the strength of the conclusion.

Major points:

The authors frequently show scans of gels and rely on reprobing the same blot to provide evidence that two things are of similar sizes. In order to make the data interpretable, could the authors ensure the following things:

- All axis and labels are labelled clearly so that it can be determined what each lane is. For example, the blots in figure 1 are labelled P, then with numbers. What do these numbers mean (or P for that matter)?

- All blots should have the size markers listed on them to allow direct comparison of the different sizes of the bands. It should also list what the numbers mean (eg. Are they kDa)?

- All blots should be shown in full (uncropped), either in the figure or collectively in a supplementary figure. Any cropped blots should be cropped correctly to ensure that the information the authors want to display is not lost (Eg. Fig 2A)

- Any arrows etc should point directly at the band(s) rather than in the general vicinity of the band for clarity

For experiments where the authors suggest that NLRP3 is unable to be detected by the antibody specific for the NLRP3 pyrin domain due to blockage of the binding site by oxDNA, can the authors please reprobe these blots with a different antibody against NLRP3, either a polyclonal antibody or one targeting a different domain, to demonstrate that NLRP3 is present and at the size the authors suggest.

To ensure that the binding of oxDNA or DNA to NLRP3 that the authors observe is specific to NLRP3, could the authors please test oxDNA binding to another NLR, perhaps NLRP1. Indeed, looking at whether the IDRs exist in other NLRs and whether they also have residues that match the DNA binding proteins highlighted in this study would be informative and should be done to demonstrate that these residues are specific for NLRP3.

We appreciated the work the authors have put in to compare the structure of NLRP3 with that of other DNA or nucleotide binding proteins to determine which residues might be required for DNA binding, which was quite interesting. However, this data is not then followed up on experimentally. Could the authors please perform site directed mutagenesis of the identified residues in NLRP3 suggested to be required for DNA binding and repeat the oxDNA binding assay to determine if these predictions are

correct. Such information would be advantageous as it could also then be translated into cell-based assays assessing NLRP3 activation in the future.

The authors produce recombinant NLRP3 for their study, which we appreciate is quite an effort. It was reported by Hochheiser et al that they see two elution peaks when they purify NLRP3, and that one of these peaks represents the oligomeric form. Can the authors please provide the data from the size exclusion column and highlight which fractions of NLRP3 were used for their assay. It would also be appreciated if the authors can include a characterisation of the oligomerisation state of their purified NLRP3 by native page.

The authors claim that the oxDNA binds to the Pyrin domain, or the intrinsically disoredered region of NLRP3 distal to the PYD domain. Can the authors please generate a truncated version of NLRP3 containing only this domain, and a version lacking this domain, and demonstrate that binding of oxDNA is specific to the IDR? It would be interesting to know whether the domains outside of this region also participate in oxDNA binding.

Previous studies by Chen et al have implicated PI(4)P as an interaction scaffold for NLRP3, and the data presented in this study suggests that oxDNA may bind to the same region as PI(4)P. Could the authors please perform a competitive binding assay to determine whether the binding site is shared between these two potential ligands?

Minor points:

- There are frequent typos and missed words in the manuscript that need to be corrected, including in some headings (eg. NLRP3 pyrin shares protein fold with human glycosylase, which should read "The NLRP3 pyrin domain shares….). Whilst it is understandable that these were missed in the preparation of the manuscript, they detract from the study and make it harder to understand. Could these please be fixed throughout the manuscript.
- In figure 1, it is unclear why NLRP3 elutes at both 0.2 and 1M NaCl. NLRP3 does not elute from the beads at 0.2M NaCl in the experiment using non-oxidised DNA, suggesting that elution of NLRP3 at it is not just non-specific binding/elution. Could the authors please provide an explanation for this? It seems odd that NLRP3 would elute at both ends of the spectrum but not in the concentrations between them. It is also concerning that the amount of NLPR3 in the flow through of the oxDNA experiment is much, much greater than that of the non-oxDNA experiment, suggesting difference in the two either in the amount of NLRP3 used or in the level of exposure. The authors need to either find a control that demonstrates that equal amounts of NLRP3 were used in the experiment and provide equal exposure for these blots or, alternatively, multiple exposures of both blots where the size and intensity of the FT bands are comparable between the different conditions.
- In figure 2 the blot in 2A is cut off, making it impossible to interpret. The experiment would also be strengthened by a comparison to an immunoblot of the NLRP3 still bound to the beads, which should reduce as more NLRP3 is eluted (or, if there is sufficient NLRP3, stay constant). Either way this is an important control for the amount of protein in each sample.
- To increase the strength of the data in figure 5 regarding the competitive binding site for oxDNA and the pyrin domain specific antibody, the authors should perform an SPR experiment comparing the two binding to NLRP3 in the presence or absence of each other.
- As mentioned earlier, I am concerned about the strength of the conclusions drawn in the discussion when compared to the quality of the data supporting it. It would be good if the authors could rephrase some of their discussion points to allow for alternative interpretations of the data, as well as reduce the strength of the conclusion where it is not entirely supported. Eg. Line 259 where the authors speculate that the 90bp DNa triggers oligomer formation of NLRP3. There is insufficient evidence in the study as it stands at the moment that the change in the size of the DNA band is due to NLRP3. To

conclude this the authors need to demonstrate, using an antibody that is not blocked by the binding of DNA
- The authors discuss ROS generation by NADPH oxidases being involved in NLRP3 activation. To my knowledge this is a controversial topic as a paper examining a knock out of the enzymatic domain of the complex showed no effect on NLRP3 activation. The involvement of mitochondrial ROS is also controversial, and in light of the recent study from the Chandal group demonstrating that mtROS is not a requirement for NLRP3, also questions the data behind this. These papers should be included in the discussion concerning ROS generation and NLRP3 activation.

Reviewer #2 (Remarks to the Author):

In the present study, Wang et al. attempt to demonstrate a direct interaction between oxidized mitochondrial DNA and NLRP3 protein and to identify protein domains responsible for this DNA binding. Their work is based on prior observations that NLRP3 bind to oxidized mitochondrial DNA either directly or indirectly (for example, Immunity 36: 401-414 (2012)). Wang et al. use EMSA (Fig. 1A-C) to show direct or indirect interaction between oxidized mitochondrial DNA and NLRP3 protein, and three sets of experiments (pull-down (Fig. 1D), pull-down with competition (Fig. 2A-C), thermal shift (Fig. 2D)) to demonstrate direct binding of oxidized mitochondrial DNA to NLRP3. Authors then use cryo-EM structure (Fig. 3A) and protein sequence alignment to C. acetobutylicum glycosylase (Fig. 3B-D) to build a hypothesis that a large positive surface of NACHT domain in NLRP3 is important for interaction with 8-oxoguanine DNA. Lastly, the authors use monoclonal antibody against pyrin domain of NLRP3 to show that a different domain, pyrin domain, of NLRP3 is also important for interaction with oxidized mitochondrial DNA.

Overall, the binding data (Fig. 1, 2) need to be explained in more detail or clarified to convince the readers that there is a direct binding between oxidized mitochondrial DNA and NLRP3 protein. Moreover, the key amino acid residues identified from sequence alignment need to be verified with mutagenesis for the information presented in Fig. 3 and 4 to be useful for the readers.

<B>The following are specific points regarding the data in main figures:</B>

<B>Concerning Fig. 1A-C (EMSA)</B>
1) In line 96, the authors state that “there was a notable shift that clearly increased with protein concentration in the same vicinity where NLRP3 migrates”. Since there are many bands (or smears) in anti-biotin-DNA blot (lefthand side), it is not clear from the image where the shift occurred, especially for the yellow arrow. For WT NLRP3, there seems to be an increased intensity of smear for the yellow arrow starting with lane “3” whereas NLRP3 is detected starting in lane “5”. Also, the anti-biotin-DNA band is strongest for lane “6” (WT NLRP3) but anti-NLRP3 band is highest in lane “8” and “9”. In addition, the huge loss of anti-biotin-DNA signal in red arrow (lower molecular weight) for WT NLRP3 is not paralleled by increase in anti-biotin-DNA signal in yellow arrow (higher molecular weight corresponding to NLRP3 protein). It would be helpful if the authors explain in more detail how the readers should interpret the data and present a more closeup image of the bands the authors wish to show.

2) For Fig. 1, the annotations need to be explained in the figure legend. For example, what does “P” stand for and what are the concentrations of NLRP3 extract corresponding to numbers 1 to 10? What are the molecular weight markers for Fig. 1A-1C?

3) For Fig. 1B and 1C anti-NLRP3 blot, two blots are presented as one blot. The entire blot image also

needs to be in the supplementary figure.

<B>Concerning Fig. 1D (pull-down with oxidized and non-oxidized DNA)</B>
4) Why are NLRP3 protein eluted at both 0.2M NaCl (low salt) and 1.0M NaCl (high salt) in Fig. 1D? Could the authors offer explanation in the result or discussion?

5) What does “FT” stand for in Fig. 1D? Can authors indicate in the figure legend?

<B>Concerning Fig. 2A-C (pull-down with oxidized and non-oxidized mitochondrial DNA)</B>
6) In Fig. 2A, there are 10 columns of bands across three rows (WT-oxidized, WT-Non-oxidized, C790T (L266F) oxidized). What does each column represent?

7) In Fig. 2A-2C, are the biotinylated mitochondrial DNA oxidized or are the competitor DNA oxidized? Can authors clarify in the figure legend or results section?

8) In the PDF version of the manuscript, the middle band (Wt-Non-oxidized) in Fig. 2A looks to be truncated at the top. The space above the band needs to be shown.

9) Do the data in Fig. 2B contradict with data in Fig. 1D? For the WT, shouldn’t the IC50 for the oxidized mitochondrial DNA (247nM) be lower than non-oxidized mitochondrial DNA (4.8nM)? Can the authors offer explanation in results or discussion?

10) In Line 246, authors state that “this might explain why FCAS is more sensitive to external activators than normal NLRP3”. However, wildtype NLRP3 has lower IC50 for oxidized mitochondrial DNA (4.8nM) compared to IC50 of mutant NLRP3 for oxidized mitochondrial DNA (8.1nM). Can the authors offer explanation in results or discussion?

<B>Concerning Fig. 2D (thermal shift assay)</B>
11) For data in Fig. 2D, are there plots such as d(RFU)/dT value (y-axis) versus temperature (x-axis) that were used to derive Tm? It would be helpful to have such plot for the SYPRO Orange in the supplementary material.

12) In Fig. 2D, why do non-transfected cells have higher Tm than transfected? Does this mean that other proteins (non-NLRP3 protein) in non-transfected cells are responsible for most of the SYPRO orange dye signal? If this is so, if you express NLRP3 under such environment (high background), how can you interpret the Tm signal? Please explain in results or discussion.

13) In Fig. 2D, the concentration of 8-oxo-dGTP (mg/ml) are indicated for transfected whereas the rest of the data are labeled in dilution factor. Also, are the data for the blue and green bars corresponding to “transfected”? The data label needs to be consistent and complete.

14) What was the statistical test used to determine the p-value?

<B>Concerning Fig. 3A (analysis of cryo-EM structure)</B>
15) In line 168, the authors state that “large positive surface spans from the NACHT domain and is continuous through the linker region which is between the NACHT and pyrin domains”. Are the authors trying to state that “large positive surface” could potentially be the binding site for oxidized mitochondrial DNA? If so, it would be helpful if the authors stated as such.

<B>Concerning Fig. 3B and Fig. 4A (alignment with C. acetobutylicum glycosylase and human glycosylase)</B>

16) In Fig. 3B, the authors find sequence alignment between NACHT domain of NLRP3 and C. acetobutylicum glycosylase. On the other hand, in Fig. 4A, authors find sequence alignment between pyrin domain of NLRP3 and human glycosylase. Are authors stating that both NACHT and pyrin domain are important for oxidized mitochondrial DNA binding? If so, this would span a large region of NLRP3. The authors would need to use mutagenesis of key residues in the NLRP3 construct followed by assays used in previous figures to demonstrate specific sites that are critical for the binding oxidized mitochondrial DNA.

<B>Concerning Fig. 5</B>
17) In Fig. 5A, 5B, if the authors found disappearance of anti-NLRP3 band with higher concentration of oxidized mitochondrial DNA using monoclonal antibody specific for pyrin domain of NLRP3 (AdipoGen), can the authors show retention of anti-NLRP3 band using different antibody that recognize other domains of NLRP3? Otherwise, the statement in discussion section for line 233-235, "we report the first study to … map the binding to the pyrin domain using a monoclonal antibody" cannot be validated.

18) What do green and purple arrows represent?

19) If authors found disappearance of anti-NLRP3 band upon increased concentration of oxidized mitochondrial DNA using monoclonal NLRP3 antibody specific for pyrin domain, and this was the only antibody used throughout the study, what does this mean for the data in rest of the figures? Can authors explain in discussion that specificity of NLRP3 antibody (pyrin domain) does not affect data presented in Fig. 1 and 2?

20) Can the authors trim images in Fig. 5 and show full images as supplementary figure? The air bubbles in Fig. 5B anti-biotin DNA blot would look "too raw" for main figure panel.

<B>The following are minor technical points:</B>
1) Line 12: None of the authors are affiliated with #3 (Department of Pharmacology, UCSD)
2) Line 66: There should be a comma between "monosodium urate (MSU)" and "calcium pyrophosphate dihydrate (CPPD)"
3) Line 69: What do MWS, CINCA/NOMID stand for?
4) Line 72: In the sentence, "many that they trigger a common cellular event", what does "many" and "they" refer to?
5) Line 104: For "data not shown", it is important to show the data since the EMSA data are difficult to understand. Can the authors at least provide the data in supplementary figure?
6) Line 229: For "data not shown", can authors provide the data in supplementary figure?
7)Line 249: For "D-loop mtDNA with oxidized guanine was synthesized by IDT", which region did the DNA correspond to specifically? For example, for 20bp and 90bp sequences used in Fig. 5, which regions were used? For the DNA used in Fig. 1A-C EMSA, which region was used?
8) Line 258: In the statement, "we show NLRP3 can bind both 20bp and 90bp oxidized DNA (Fig. 10)", there is no Figure 10 in the manuscript.
9) Line 266: In the statement, "we show the pyrin domain can bind oxidized mtDNA and that the NACHT domain is also poised for an interaction with 8-oxoguanine DNA with its positive surface and homology to CacOgg (Fig. 6), there is no Figure 6 in the paper.
10) In the methods section, the statistical method is missing.
11) For gel images such as Fig. 3S, blots from different images (molecular weight) are stuck onto another image. Can authors make sure that the image integrity guideline in Communications Biology is followed?
https://www.nature.com/commsbio/editorial-policies/image-integrity

Reviewer #3 (Remarks to the Author):

In this manuscript the authors investigate the direct binding of Nlrp3 to non-oxidized and oxidized mtDNA. The use an FCAS mutant to show differential binding in a disease mutant of Nlrp3. The authors furthermore try to compare Nlrp3 to other proteins characterized to bind oxo mtDNA, glycosylases and predict a similar structure capable to bind DNA. The region between the PYD and NACHT domain of Nlrp3 is predicted to be disordered and positively charged, potentially aiding in DNA binding.

Several publications have recently claimed direct binding of mtDNA to Nlrp3 as an activation signal. This publication tries to show direct binding of Nlrp3 with biochemical assays and further support by bioinformatic methods. Direct bindig of mtDNA was claimed by several publications before but could never been convincing shown biochemically.

However, in the current version the manuscript misses further experiments to confirm this finding and some results within the paper seem to disprove each other, leaving it overall confusing and a mix of different observations.

Major points:

1. Does Figurer 1 show an EMSA with purified Nlrp3 or cellular extract with different expression levels of Nlrp3? The methods section only mentions EMSAs with purified Nlrp3, the results section speaks about cells. If so, what where the transfection conditions (amounts?). This should be added to the Methods section.
2. The two chosen CAPS mutations are well known mutations in the NACHT domain of Nlrp3 and far away from any of the proposed binding sites but interestingly seem to still abolish oxo mtDNA binding. Did they show the same behaviour when using the Dynabeads pulldown assay?
3. In the description of the pulldown experiments for Figure 2 it seems to miss the step where the Nlrp3 protein is added to the mix.
4. In Fig 2C the IC50 of the C790T mutation for non-oxidised mtDNA is determined in the low nanomolar range, yet the authors did not observe any effect in the EMSAs in Figure 1. Is there any explanation for this?
5. The authors describe the recent decamr/do-decamer Cryo-EM structure of Nlrp3 and identify the polybasic patch in the linker region between the PYD and NACHT as a potential DNA binding site. It should be mentioned here that this region has recently been identified to bind lipids and essential for the interaction of Nlrp3 with membranes, which are, as DNA is, negatively charged and at least the latter part of the linker has been shown to be essential in the sensing of K+ efflux.
6. The sequence homology is very low and only very few patches between CacOgg and Nlrp3 are similar and are also not positively charged, making it doubtful they would be important in direct DNA recognition. Furthermore, they are well within the NACHT domain and outside of the disordered linker domain. In comparison between Fig 3A and Fig 3C I also note that the green arrow in 3C points to a short IDR but also points to an apparently negatively charged patch on the surface of the Nlrp3 protein.
7. The sequence homology between Hogg1 and Nlrp3 is also not high and some of the helices seem to be quite different between the PYD of Nlrp3 and Hogg1. If the authors want to claim any sequence homology here, some RMSD values would enhance the argument (e.g. results of Matchmaker).
8. The described CAPS mutations are interesting but many of them seem to not directly change the surface charge of the PYD and thus making me wonder if they would be having any effect on the binding of ox or non-ox mtDNA?

9. In Figure 5 the authors claim that they used the PYD domain. But they include the C1058T mutant of Nlrp3. C1058T mutation leads to a point mutation of L353P. This is within the NACHT domain and not the PYD, which the authors claim to be used with residues 1-93. The EMSAs in Figure 1 looked much better, I wonder why they don't repeat these with the PYD domains, especially with the mutations described in Figure 4? This would really add to the story and make a good connection.

Minor points:

- The nomenclature throughout the manuscript should be corrected to the correct nomenclature for all protein and gene names.
- The usage of concentrations either in g/ml or μM is confusing, please use Greek letters when necessary.
- The protein purification protocol is not complete, it misses the final elution and assumingly a concentration step. It would be good to include an SDS-PAGE of the purification in the supplementals to confirm purity of the purification.
- Figure S1 shows two blots for ERK1/2, why? It is also confusing to rename Nlrp3 to its historic CIAS1 name and not mention it in the figure legend.

Dear Reviewers,

We have prepared a revised version of our paper “Characterization of a Direct Ligand Interaction that Mediates NLRP3 Inflammasome Activation”, considering the comments of all referees. A detailed list of the responses to the comments is appended below.

Reviewer 1.

This reviewer was appreciative of the effort involved in our work, and we address the following recommendations below.

Major points:

1. *The authors frequently show scans of gels and rely on reprobing the same blot to provide evidence that two things are of similar sizes. In order to make the data interpretable, could the authors ensure the following things:*

- All axis and labels are labelled clearly so that it can be determined what each lane is. For example, the blots in figure 1 are labelled P, then with numbers. What do these numbers mean (or P for that matter)?

P stands for protein alone. The numbers represent different lanes of 1:2 dilutions of protein which are increasing from left to right. We have updated the labels for this figure.

- All blots should have the size markers listed on them to allow direct comparison of the different sizes of the bands. It should also list what the numbers mean (eg. Are they kDa)?

The size markers are in kDa and have been updated in the figures.

- All blots should be shown in full (uncropped), either in the figure or collectively in a supplementary figure. Any cropped blots should be cropped correctly to ensure that the information the authors want to display is not lost (Eg. Fig 2A)

Figure 2A has been re-uploaded to show the full-rendered image. The full gel has also been added as a supplemental figure.

- Any arrows etc should point directly at the band(s) rather than in the general vicinity of the band for clarity

All arrows now point directly at bands.

2. *For experiments where the authors suggest that NLRP3 is unable to be detected by the antibody specific for the NLRP3 pyrin domain due to blockage of the binding site by oxDNA, can the authors please reprobe these blots with a different antibody against NLRP3, either a polyclonal antibody or one targeting a different domain, to demonstrate that NLRP3 is present and at the size the authors suggest.*

The reviewer is correct that a different antibody should be able to restore lost intensity. We should clarify that the gel is run under native conditions and contains a mixture of bound and unbound NLRP3. The NLRP3 unbound to DNA is detected with the monoclonal antibody used in this study. NLRP3 bound to DNA is not detected. This results in a shadow effect with the unbound NLRP3 surrounding or immediately above the DNA bound. We agree it would be

useful to use an antibody that removes this shadow effect and concurrently shows the bound NLRP3. As we were instructed elsewhere to remove the pyrin domain and repeat the binding assay, we discovered that NLRP3 lacking the pyrin domain can also bind DNA (Figure S7). We further show that an antibody against the NACHT domain can detect full-lenth NLRP3 bound to DNA as well as the NLRP3 lacking the pyrin domain (Figure 6). We'd also highlight our Blue-Native western shows darker intensity when DNA is not added. This further illustrates the difficulty for the monoclonal antibody to bind when DNA is present.

3. *To ensure that the binding of oxDNA or DNA to NLRP3 that the authors observe is specific to NLRP3, could the authors please test oxDNA binding to another NLR, perhaps NLRP1. Indeed, looking at whether the IDRs exist in other NLRs and whether they also have residues that match the DNA binding proteins highlighted in this study would be informative and should be done to demonstrate that these residues are specific for NLRP3*

The reviewer is correct that we do not know if the DNA binding is specific for NLRP3 compared to other NLR's. In pairwise alignment of NLRP1 to bacterial glycosylase most sequence similarity did map to the NAHCT domain. However, none of the residues important in binding oxidized DNA shared with NLRP3 were present in NLRP1 (Figure S10). Pairwise alignment of human glycosylase with NLRP1 mapped to the NACHT domain but did not share the same amino acids referenced in this paper. After pairwise alignment of NLRP1-14 pyrin domain to hOGG1, we have identified several residues important in binding oxidized DNA that are completely shared only between NLRP3 and hOGG1 (Figure S10).

As requested, we have examined whether IDR's exist in other NLR's and if they have matching residues implicated in this study. NLRP3's IDR was about half way from the linker region all the way up to the NACHT domain. The only NLR's with intrinsically disordered regions in the same vicinity were NLRP 6, 10, and 12. None of these NLR's shared all the residues identified herein (Figs. 3, 4, and Fig S. 10).

4. *We appreciated the work the authors have put in to compare the structure of NLRP3 with that of other DNA or nucleotide binding proteins to determine which residues might be required for DNA binding, which was quite interesting. However, this data is not then followed up on experimentally. Could the authors please perform site directed mutagenesis of the identified residues in NLRP3 suggested to be required for DNA binding and repeat the oxDNA binding assay to determine if these predictions are correct. Such information would be advantageous as it could also then be translated into cell-based assays assessing NLRP3 activation in the future.*

We suggest that the reviewer is asking for a lot more follow-up work than we can reasonably attempt at this stage. This paper represents a huge amount of work by a number of people, but it is by no means the end of the story. We will be pursuing the questions raised by the reviewer in the future; these will require experimental design quite different from what we have done so far.

5. *The authors produce recombinant NLRP3 for their study, which we appreciate is quite an effort. It was reported by Hochheiser et al that they see two elution peaks when they purify NLRP3, and that one of these peaks represents the oligomeric form. Can the authors please provide the data from the size exclusion column and highlight which fractions of NLRP3 were used for their assay. It would also be appreciated if the authors can include a characterisation of the oligomerisation state of their purified NLRP3 by native page.*

The NP-40 in gel filtration buffer exhibits strong absorbance at 280nm. Nonetheless, we have provided SDS PAGE gel data from the size exclusion column to show the full-length NLRP3 used in this study elutes in the void volume using our HiLoad 16/600 Superose 6 column (Fig. S8). The Blue native western included in Fig. S5 shows a complex around 1200 kDa which is consistent with the oligomerized form of NLRP3.

6. *The authors claim that the oxDNA binds to the Pyrin domain, or the intrinsically disoredered region of NLRP3 distal to the PYD domain. Can the authors please generate a truncated version of NLRP3 containing only this domain, and a version lacking this domain, and demonstrate that binding of oxDNA is specific to the IDR? It would be interesting to know whether the domains outside of this region also participate in oxDNA binding.*

We thank the reviewer for this question which increases the impact of this study. Site-directed mutagenesis was used to create a construct lacking the pyrin domain $NLRP3_{(94\text{-}1034)}$ and one that added a stop codon after the pyrin domain $NLRP3_{1\text{-}93}$. We attached biotinylated oxidized or non-oxidized DNA to streptavidin Dyna beads and found we could pulldown $NLRP3_{(94\text{-}1034)}$ with both forms of DNA (Fig. S7). We analyzed the isolated pyrin domain $NLRP3_{1\text{-}93}$ by binding to biotinylated oxidized/non-oxidized DNA and subsequently competing the protein off the beads with several concentrations non-biotinylated DNA. We analyzed the remaining bound fraction. Interestingly, $NLRP3_{1\text{-}93}$ showed very little binding to non-oxidized DNA and was barely visible when looking at the bound fraction. Nonetheless, the pyrin domain $NLRP3_{1\text{-}93}$ had much stronger preference for oxidized DNA. So, while the pyrin deleted construct $NLRP3_{(94\text{-}1034)}$ has some propensity to bind both oxidized and non-oxidized DNA, the pyrin domain $NLRP3_{1\text{-}93}$ is more specific for oxidized DNA.

7. *Previous studies by Chen et al have implicated PI(4)P as an interaction scaffold for NLRP3, and the data presented in this study suggests that oxDNA may bind to the same region as PI(4)P. Could the authors please perform a competitive binding assay to determine whether the binding site is shared between these two potential ligands?*

The previous studies by Chen (Chen et. Al 2018, *Nature*) demonstrate that PI(4)P interacts with the KMKK motif of NLRP3 (residues 131-134) which is outside the pyrin domain and preceding the NACHT domain. To answer if oxDNA is binding to the same region, we used site-directed mutagenesis to add a stop codon after the pyrin domain $NLRP3_{1\text{-}93}$ which does not include the KMKK sequence. $NLRP3_{1\text{-}93}$ preferentially binds oxidized DNA (Fig. 6). Thus, the binding site for PI(4)P and oxidized DNA is not shared between these two ligands.

Minor points:

1. There are frequent typos and missed words in the manuscript that need to be corrected, including in some headings (eg. NLRP3 pyrin shares protein fold with human glycosylase, which should read "The NLRP3 pyrin domain shares….). Whilst it is understandable that these were missed in the preparation of the manuscript, they detract from the study and make it harder to understand. Could these please be fixed throughout the manuscript.

We have edited the manuscript to state NLRP3 pyrin domain instead of NLRP3 when comparing to human glycosylase fold. We also checked for typos.

2. In figure 1, it is unclear why NLRP3 elutes at both 0.2 and 1M NaCl. NLRP3 does not elute from the beads at 0.2M NaCl in the experiment using non-oxidised DNA, suggesting that elution of NLRP3 at it is not just non-specific binding/elution. Could the authors please provide an explanation for this? It seems odd that NLRP3 would elute at both ends of the spectrum but not in the concentrations between them.

In these experiments, we are looking at the unbound fraction, or what eluted from the beads. Protein remaining on the beads was not analyzed in this study and it remains possible that the beads for non-oxidized DNA, that don't show elution at 0.2M NaCl, still have DNA bound to beads. The elution at 0.2M for oxidized DNA and not for non-oxidized DNA suggest there might be different mechanisms by which NLRP3 can bind both oxidized DNA and non-oxidized DNA. In the resubmission data, we have shown that the pyrin domain prefers oxidized DNA, while the NACHT-LRR construct (NLRP3$_{(94-1034)}$) can bind either non-oxidized or oxidized DNA. It is possible that this is the reason for variable salt elution in Figure 1D.

3. It is also concerning that the amount of NLPR3 in the flow through of the oxDNA experiment is much, much greater than that of the non-oxDNA experiment, suggesting difference in the two either in the amount of NLRP3 used or in the level of exposure. The authors need to either find a control that demonstrates that equal amounts of NLRP3 were used in the experiment and provide equal exposure for these blots or, alternatively, multiple exposures of both blots where the size and intensity of the FT bands are comparable between the different conditions.

These experiments were done at the same time with the same sample. They have similar exposure. We have no clear explanation for the difference in the appearance of the flow through (FT) for oxidized and non-oxidized. We often see higher order species of NLRP3 when the sample is not boiled compared to when it is. To the left of the protein only lane (P) in 1 figs. C and D, we included an equally loaded amount of denatured protein. In the native state, the protein forms higher order species and appears over exposed even though it's on the same membrane. Equal loading of all constructs used in this figure is illustrated in Fig. S1 as evidenced by anti-Erk1/2.

4. In figure 2 the blot in 2A is cut off, making it impossible to interpret. The experiment would also be strengthened by a comparison to an immunoblot of the NLRP3 still bound to the beads, which should reduce as more NLRP3 is eluted (or, if there is sufficient NLRP3, stay constant). Either way this is an important control for the amount of protein in each sample.

We have re-uploaded figure 2 to show the full image. There are slight deviations in bead loading in the assay as well as pipetting the beads for the gel. We have labeled figure 2 to illustrate the first lane on the blot is uneluted protein. The variation between datapoints is illustrated by y-error bars in fig. 2B-C. We also provide the initial amount of NLRP3$_{1-93}$ on the beads as the first lane in Fig. 7.

5. *To increase the strength of the data in figure 5 regarding the competitive binding site for oxDNA and the pyrin domain specific antibody, the authors should perform an SPR experiment comparing the two binding to NLRP3 in the presence or absence of each other.*

We acknowledge more detailed information regarding the binding interaction and the molecular determinants of recognition are the next steps for this study. Due to resources, we plan to address this in the near future.

6. As mentioned earlier, I am concerned about the strength of the conclusions drawn in the discussion when compared to the quality of the data supporting it. It would be good if the authors could rephrase some of their discussion points to allow for alternative interpretations of the data, as well as reduce the strength of the conclusion where it is not entirely supported. Eg. Line 259 where the authors speculate that the 90bp DNa triggers oligomer formation of NLRP3. There is insufficient evidence in the study as it stands at the moment that the change in the size of the DNA band is due to NLRP3. To conclude this the authors need to demonstrate, using an antibody that is not blocked by the binding of DNA.

We have verified that an antibody not blocked by binding of DNA is able to restore NLRP3 intensity (Figure 6). We have adjusted ambiguous conclusions.

7. The authors discuss ROS generation by NADPH oxidases being involved in NLRP3 activation. To my knowledge this is a controversial topic as a paper examining a knock out of the enzymatic domain of the complex showed no effect on NLRP3 activation. The involvement of mitochondrial ROS is also controversial, and in light of the recent study from the Chandal group demonstrating that mtROS is not a requirement for NLRP3, also questions the data behind this. These papers should be included in the discussion concerning ROS generation and NLRP3 activation.

We have deleted the reference to NADPH oxidases. We instead mention newly synthesized mt-DNA that gets exposed to ROS produced from loss of mitochondrial membrane potential related to calcium and potassium flux (Grung et al, 2015 and Zhong et al 2016).

Reviewer 2.

This reviewer was appreciative of our work.

The following are specific points regarding the data in main figures:

Concerning Fig. 1A-C (EMSA)

1) In line 96, the authors state that “there was a notable shift that clearly increased with protein concentration in the same vicinity where NLRP3 migrates”. Since there are many bands (or smears) in anti-biotin-DNA blot (lefthand side), it is not clear from the image where the shift occurred, especially for the yellow arrow. For WT NLRP3, there seems to be an increased intensity of smear for the yellow arrow starting with lane “3” whereas NLRP3 is detected starting in lane “5”.

The right and left sides of Figure 1 are from the same membrane that has been reprobed with 2 different antibodies at exposures required for the antibody. It should also be noted that this EMSA was done with lysate and there are several shifts. We note that one of the observed shifts

is in the same vicinity as where NLRP3 antibody can detect NLRP3. We didn't find out until Figure 5 that under native conditions, the antibody can only detect unbound NLRP3. So, any NLRP3 visible in Figure 1(A-C) is really unbound to DNA.

Also, the anti-biotin-DNA band is strongest for lane "6" (WT NLRP3) but anti-NLRP3 band is highest in lane "8" and "9". In addition, the huge loss of anti-biotin-DNA signal in red arrow (lower molecular weight) for WT NLRP3 is not paralleled by increase in anti-biotin-DNA signal in yellow arrow (higher molecular weight corresponding to NLRP3 protein). It would be helpful if the authors explain in more detail how the
readers should interpret the data and present a more closeup image of the bands the authors wish to show.

The reviewer is correct that the loss of free DNA and subsequent shift is not exactly correlated with the anti-NLRP3 signal. At first, we thought that this was because other proteins in the lysate were also binding the DNA, which is true. But in Figs. 5 and 6, we discovered the monoclonal antibody against the NLRP3 pyrin domain has a problem detecting NLRP3 bound to DNA. We illustrate that a different antibody against the NACHT domain can restore NLRP3 intensity.

2) For Fig. 1, the annotations need to be explained in the figure legend. For example, what does "P" stand for and what are the concentrations of NLRP3 extract corresponding to numbers 1 to 10? What are the molecular weight markers for Fig. 1A-1C?

We have updated the annotations. 'P' stands for Protein without DNA. Numbers represent different lanes with increasing concentrations of protein extract from left to right. We do not show a protein ladder in Figs 1A-C. The lane that resembles a ladder is non-specific labeling of protein in the absence of DNA by anti-biotin.

3) For Fig. 1B and 1C anti-NLRP3 blot, two blots are presented as one blot. The entire blot image also needs to be in the supplementary figure.

Uncropped images of 1B and 1C have been added uncropped (Figure S11).

Concerning Fig. 1D (pull-down with oxidized and non-oxidized DNA)
4) Why are NLRP3 protein eluted at both 0.2M NaCl (low salt) and 1.0M NaCl (high salt) in Fig. 1D? Could the authors offer explanation in the result or discussion?

This has been addressed in Reviewer 1 minor points #2.

5) What does "FT" stand for in Fig. 1D? Can authors indicate in the figure legend?

We have updated the figure legend. "FT" stands for flow through.

Concerning Fig. 2A-C (pull-down with oxidized and non-oxidized mitochondrial DNA)
6) In Fig. 2A, there are 10 columns of bands across three rows (WT-oxidized, WT-Non-oxidized, C790T (L266F) oxidized). What does each column represent?

Each column represents an increasing amount of competitor DNA, increasing from left to right.

7) In Fig. 2A-2C, are the biotinylated mitochondrial DNA oxidized or are the competitor DNA oxidized? Can authors clarify in the figure legend or results section?

The hot and cold ligands are the same in these experiments, except for being biotinylated or not.

8) In the PDF version of the manuscript, the middle band (Wt-Non-oxidized) in Fig. 2A looks to be truncated at the top. The space above the band needs to be shown.

We will double check this image uploads properly.

9) Do the data in Fig. 2B contradict with data in Fig. 1D? For the WT, shouldn't the IC50 for the oxidized mitochondrial DNA (247nM) be lower than non-oxidized mitochondrial DNA (4.8nM)? Can the authors offer explanation in results or discussion?

The data in Figure 1D uses salt elution and analyzes what was eluted from the beads. Figure 2B analyzes the beads directly (bound fraction) and elution was done with competitor DNA.

10) In Line 246, authors state that "this might explain why FCAS is more sensitive to external activators than normal NLRP3". However, wildtype NLRP3 has lower IC50 for oxidized mitochondrial DNA (4.8nM) compared to IC50 of mutant NLRP3 for oxidized mitochondrial DNA (8.1nM). Can the authors offer explanation in results or discussion?

We reported wildtype NLRP3 has an IC50 of 4nM for non-oxidized DNA, not oxidized. Thus, our initial rational is correct.

Concerning Fig. 2D (thermal shift assay)
11) For data in Fig. 2D, are there plots such as d(RFU)/dT value (y-axis) versus temperature (x-axis) that were used to derive Tm? It would be helpful to have such plot for the SYPRO Orange in the supplementary material.

The Tm's were derived directly by the RT-PCR machine software and directly transferred to PRISM software to make the plot.

12) In Fig. 2D, why do non-transfected cells have higher Tm than transfected? Does this mean that other proteins (non-NLRP3 protein) in non-transfected cells are responsible for most of the SYPRO orange dye signal? If this is so, if you express NLRP3 under such environment (high background), how can you interpret the Tm signal? Please explain in results or discussion.

The reviewer is correct that there is a difference in baseline for non-transfected vs transfected cells. Because of this, all comparisons are done only to respective condition without the drug. We are not comparing transfected and un-transfected for statistical analysis, just transfected.

13) In Fig. 2D, the concentration of 8-oxo-dGTP (mg/ml) are indicated for transfected whereas the rest of the data are labeled in dilution factor. Also, are the data for the blue and green bars corresponding to "transfected"? The data label needs to be consistent and complete.

We have listed dilution factor for all treatments.

14) What was the statistical test used to determine the p-value?

One-way ANOVA. We have added this to the manuscript.

Concerning Fig. 3A (analysis of cryo-EM structure)
15) In line 168, the authors state that "large positive surface spans from the NACHT domain and

is continuous through the linker region which is between the NACHT and pyrin domains". Are the authors trying to state that "large positive surface" could potentially be the binding site for oxidized mitochondrial DNA? If so, it would be helpful if the authors stated as such.

The reviewer is correct that the large positive surface could potentially be a binding site for DNA. We have explicitly stated this in the revised version.

Concerning Fig. 3B and Fig. 4A (alignment with C. acetobutylicum glycosylase and human glycosylase)

16) In Fig. 3B, the authors find sequence alignment between NACHT domain of NLRP3 and C. acetobutylicum glycosylase. On the other hand, in Fig. 4A, authors find sequence alignment between pyrin domain of NLRP3 and human glycosylase. Are authors stating that both NACHT and pyrin domain are important for oxidized mitochondrial DNA binding? If so, this would span a large region of NLRP3. The authors would need to use mutagenesis of key residues in the NLRP3 construct followed by assays used in previous figures to demonstrate specific sites that are critical for the binding oxidized mitochondrial DNA.

The reviewer is correct that there are similarities to glycosylase in both the NACHT and pyrin domain. To further address the point of Reviewer #2, we have used site-directed mutagenesis to generate: 1) NLRP3 pyrin domain only (Fig. 7) and 2) NLRP3 lacking the pyrin domain (Fig. S7). We show that both constructs can bind oxidized DNA. The pyrin domain alone, which has largely negative electrostatic surface potential, has preference for oxidized DNA, while the NACHT domain can interact with either.

Concerning Fig. 5

17) In Fig. 5A, 5B, if the authors found disappearance of anti-NLRP3 band with higher concentration of oxidized mitochondrial DNA using monoclonal antibody specific for pyrin domain of NLRP3 (AdipoGen), can the authors show retention of anti-NLRP3 band using different antibody that recognize other domains of NLRP3? Otherwise, the statement in discussion section for line 233-235, "we report the first study to … map the binding to the pyrin domain using a monoclonal antibody" cannot be validated.

The reviewer is correct that anti-NLRP3 band disappears with increased DNA concentration (Figs. 5a and 5b). We have shown the anti-NLRP3 can reappear when a monoclonal antibody against the NACHT domain is used (Fig. 6).

18) What do green and purple arrows represent?

The purple arrow represents one form of NLRP3, where the green arrow represents a further oligomeric form of NLRP3 bound of DNA, supporting our hypothesis that DNA binding induces NLRP3 oligomerization. We better describe these arrows in our recent update of the manuscript

19) If authors found disappearance of anti-NLRP3 band upon increased concentration of oxidized mitochondrial DNA using monoclonal NLRP3 antibody specific for pyrin domain, and this was the only antibody used throughout the study, what does this mean for the data in rest of the figures? Can authors explain in discussion that specificity of NLRP3 antibody (pyrin domain) does not affect data presented in Fig. 1 and 2?

The reviewer is correct that in native EMSA conditions the NLRP3 monoclonal antibody against the pyrin domain cannot recognize NLRP3 bound to oxidized DNA (Fig. 5). This means that in native conditions any protein detected in the western blot is unbound to DNA. This is the reason for the shadow effect around the protein (Fig. 5). The main difference between Figs. 1 and 2 is that figure 2 samples are boiled in SDS loading buffer and analyzed under denaturing conditions. Figure 1 samples are not boiled and analyzed with Tris-Glycine gels without SDS or beta-mercaptoethanol. The denaturing conditions in Figure 2 would free up NLRP3 to be properly detected by the monoclonal antibody against the pyrin domain.

The main take home message for figure 1 was that the EMSA shift was in the same vicinity as NLRP3 which was done with lysate. The purpose of subsequent figures was to identify direct binding of NLRP3 to mitochondrial DNA using purified protein. The gel in figure 1 is under native conditions with lysate and likely contains a mixture of oligomeric, monomeric, and multimeric states of NLRP3 unbound to DNA and possibly other unknown proteins. It's important for us to note that when we first did this with purified protein, we could detect shifts in the EMSA's but could not see any NLRP3 at all. It was only when we increased the concentration or amount of NLRP3 in the assay that we could see labeling of unbound NLRP3 which created a shadow around the observed DNA in the shift.

20) Can the authors trim images in Fig. 5 and show full images as supplementary figure? The air bubbles in Fig. 5B anti-biotin DNA blot would look "too raw" for main figure panel.
These images have been trimmed.

The following are minor technical points:

1) Line 12: None of the authors are affiliated with #3 (Department of Pharmacology, UCSD)

We have removed that affiliation.

2) Line 66: There should be a comma between "monosodium urate (MSU)" and "calcium pyrophosphate dihydrate (CPPD)"

We have added the comma.

3) Line 69: What do MWS, CINCA/NOMID stand for?

Familial cold-induced autoinflammatory syndrome (FCAS), Muckle Wells Syndrome (MWS), Neonatal-onset multisystem inflammatory disease (NOMID)

This has been updated.

4) Line 72: In the sentence, "many that they trigger a common cellular event", what does "many" and "they" refer to?

"many" has been deleted.

5) Line 104: For "data not shown", it is important to show the data since the EMSA data are difficult to understand. Can the authors at least provide the data in supplementary figure?

The EMSA for lysate of non-transfected cells has been added (Figure S2).

6) Line 229: For "data not shown", can authors provide the data in supplementary figure?

We have added a western blot that dually labels DNA and NLRP3 with fluorescent secondary antibodies. This is illustrated in Figure S6A.

7)Line 249: For “D-loop mtDNA with oxidized guanine was synthesized by IDT”, which region did the DNA correspond to specifically? For example, for 20bp and 90bp sequences used in Fig. 5, which regions were used? For the DNA used in Fig. 1A-C EMSA, which region was used?

The DNA sequence for the 90bp sequence is the same as that reported by Zhong et al Nature 2018. The 20bp sequence is the first 20 nucleotides from the original 90bp sequence. The both sequences are shown in Figure S6b.

8) Line 258: In the statement, “we show NLRP3 can bind both 20bp and 90bp oxidized DNA (Fig. 10)”, there is no Figure 10 in the manuscript.

We have updated this to Figure 5.

9) Line 266: In the statement, “we show the pyrin domain can bind oxidized mtDNA and that the NACHT domain is also poised for an interaction with 8-oxoguanine DNA with its positive surface and homology to CacOgg (Fig. 6), there is no Figure 6 in the paper.

We have added the binding data for the pyrin domain in Figure 7 and the modeling based on the CacOgg and the NLRP3 NACHT domain in Figure 3.

10) In the methods section, the statistical method is missing.

Statistical methods have been added.

11) For gel images such as Fig. 3S, blots from different images (molecular weight) are stuck onto another image. Can authors make sure that the image integrity guideline in Communications Biology is followed?

We have used an unstained native ladder run on a native gel that does not show up when transferred during a western. Images with stained adjacent ladders have been separated from native membrane images.

Reviewer #3

Major points:

1. Does Figurer 1 show an EMSA with purified Nlrp3 or cellular extract with different expression levels of Nlrp3? The methods section only mentions EMSAs with purified Nlrp3, the results section speaks about cells. If so, what where the transfection conditions (amounts?). This should be added to the Methods section.

Figure 1 was done with lysate and Figure 2 with purified protein. We have updated the methods to include transfection and utilization of lysate which was a 20ml culture.

2. The two chosen CAPS mutations are well known mutations in the NACHT domain of Nlrp3 and far away from any of the proposed binding sites but interestingly seem to still abolish oxo mtDNA binding. Did they show the same behaviour when using the Dynabeads pulldown assay?

The results are only similar in that CAPS mutant behaves opposite the WT. It is difficult to draw a concrete conclusion without purifying the protein, as changing how the protein binds to DNA might influence if the complex enters the gel and how it is transferred to the membrane.

3. In the description of the pulldown experiments for Figure 2 it seems to miss the step where the Nlrp3 protein is added to the mix.

We have updated the methods to include this step. Dynabeads were equilibrated according to manufacture directions, washed, incubated overnight with biotinylated oxidized or non-oxidized DNA, washed, then incubated with protein and washed.

4. In Fig 2C the IC50 of the C790T mutation for non-oxidised mtDNA is determined in the low nanomolar range, yet the authors did not observe any effect in the EMSAs in Figure 1. Is there any explanation for this?

The low nanomolar range IC50 for the mutant was shown in Fig. 2C for C790T with only oxidized DNA and not non-oxidized DNA.

5. The authors describe the recent decamr/do-decamer Cryo-EM structure of Nlrp3 and identify the polybasic patch in the linker region between the PYD and NACHT as a potential DNA binding site. It should be mentioned here that this region has recently been identified to bind lipids and essential for the interaction of Nlrp3 with membranes, which are, as DNA is, negatively charged and at least the latter part of the linker has been shown to be essential in the sensing of K+ efflux.

Based on studies in Chen et. Al 2018, *Nature*, we understand that that PI(4)P interacts with the KMKK motif of NLRP3 (residues 131-134) which is outside the pyrin domain and preceding the NACHT domain. Based on these findings, through mutagenesis we made an NLRP3 construct up to the amino acids referenced in this study, hereinafter known as $NLRP3_{(1-134)}$. Though we have successfully purified this protein, we do not see any difference in the activity of this construct compared to the pyrin only construct ($NLRP3_{(1-93)}$. We also made a construct lacking the pyrin domain, but containing this polybasic region, herein known as $NLRP3_{(94-1034)}$. We have observed the ability for this protein to bind both ox-mtDNA and non-ox-mtDNA, relatively similarly (Fig. S7).

6. The sequence homology is very low and only very few patches between CacOgg and Nlrp3 are similar and are also not positively charged, making it doubtful they would be important in direct DNA recognition. Furthermore, they are well within the NACHT domain and outside of the disordered linker domain. In comparison between Fig 3A and Fig 3C I also note that the green arrow in 3C points to a short IDR but also points to an apparently negatively charged patch on the surface of the Nlrp3 protein.

Blue is positive and red is negative. The arrow is pointing to a positive patch. It is possible that negatively charged DNA is attracted to the positive patch.

7. The sequence homology between Hogg1 and Nlrp3 is also not high and some of the helices seem to be quite different between the PYD of Nlrp3 and Hogg1. If the authors want to claim

any sequence homology here, some RMSD values would enhance the argument (e.g. results of Matchmaker).

The RMSD between 18 Å pruned atom pairs is 1.2 Å. Across all 71 pairs is 12.0 Å.

8. The described CAPS mutations are interesting but many of them seem to not directly change the surface charge of the PYD and thus making me wonder if they would be having any effect on the binding of ox or non-ox mtDNA?

It is possible the specificity of residues similar to CacOgg or hOGG1 act in concert with surface electrostatic potential. We plan to investigate this further by cryo-electron microscopy.

9. In Figure 5 the authors claim that they used the PYD domain. But they include the C1058T mutant of Nlrp3. C1058T mutation leads to a point mutation of L353P. This is within the NACHT domain and not the PYD, which the authors claim to be used with residues 1-93. The EMSAs in Figure 1 looked much better, I wonder why they don't repeat these with the PYD domains, especially with the mutations described in Figure 4? This would really add to the story and make a good connection.

We did not use the pyrin domain only for figure 5. We used full-length protein. We have improved the manuscript by adding purified pyrin domain used in Figure 7.

Minor points:

- The nomenclature throughout the manuscript should be corrected to the correct nomenclature for all protein and gene names.
- The usage of concentrations either in g/ml or µM is confusing, please use Greek letters when necessary.
- The protein purification protocol is not complete, it misses the final elution and assumingly a concentration step. It would be good to include an SDS-PAGE of the purification in the supplementals to confirm purity of the purification.

We have added an SDS gel to show how pure the samples in Figs. S8 and S9 for full-length and isolated prryin domain, respectively.

- Figure S1 shows two blots for ERK1/2, why? It is also confusing to rename Nlrp3 to its historic CIAS1 name and not mention it in the figure legend.

One blot has been imaged twice to show two exposures.

The constructs are named with the historic CIAS1 because that is exactly how they were named when they were given to us by Hal Hoffman. We have mentioned NLRP3 being synonymous with CIAS1 in the figure legend as requested.

We hope that with these changes, the paper will be acceptable for publication in *Communications Biology*.

Reviewers' comments:

Reviewer #1 (Remarks to the Author):

We thanks the authors for their updated manuscript and the efforts made to address my comments. Most of the concerns I have are covered sufficiently in the reply but I still have some things that I would like to see altered:

- Please insert the reference for the Chandal paper - there is controversy around whether ROS is required for NLRP3 activation and that should be acknowledged rather than ignored.

- I understand that time and money are limiting but in this case I'd also appreciate, in the absence of site directed mutagenesis of the DNA binding site or any cell based assay, that these limitations also be included in the discussion.

- I really appreciate the effort the authors put into looking and annotating the IDR regions of the different NLR proteins, this was interesting and will hopefully help the manuscript.

- I have some concerns about the specificity of the interaction between the DNA and NLRP3 given that it seems to bind to two different domains. I don't need this addressed but it should be taken into account or dealt with in any follow up work.

- In response to this comment "that there is no clear reason for the difference in the flow through between the non-oxidised and oxidised DNA". this is really concerning to be honest. I understand that it is a native gel, however all of these are native gels. If the system is not stable enough to give reproducible results between different gels how can the results from this experiment be trusted?

- Can units please be added to all figures, they are still missing in figure 2A for example. The concentration units are also missing (uM, nM?) and are the dotted lines the SEM?

- The start of the C790T blot for fig 2A is still cut off at the front.

- I still have concerns about figure 2A as there are no controls for:
- How much NLRP3 comes off the column when you don't have any mtDNA present?
- The total amount of NLRP3 bound to the beads prior to any elution and without this you don't know the proportion of NLRP3 eluting and whether the amount is relevant.

Reviewer #2 (Remarks to the Author):

The authors have addressed all of my concerns.

I think the readers have sufficient information to interpret the results of the paper.

The following are personal suggestions to make the paper more accessible (not requirements):

(1) It would be nice to make minor changes to ambiguous sentences such as:

Line 112 "there is no binding or that binding has occurred the complex does not enter the gel"

Line 186 "residues known to be important in binding ox-DNA that were exactly the same were"

Line 194 "Software D2P2 analysis showed several IDR prediction software programs agreed that"

Line 534 "examined for binding to ether 8-oxo-dGTP or 8-oxo-dG molecule" (ether should be either)

(2) It would also be nice to have information on how different NLRP3 constructs (1-93, 1-134, 94-1034) were generated. Authors described NLRP3 (1-93) mutagenesis in some detail (in the supplementary methods), so it would also be helpful to have information on others.

Reviewer #3 (Remarks to the Author):

I thank the authors for the extensive experimental and written responses to the points raised. Especially the addition of constructs +- PYD has improved the manuscript.

The now presented data indicates that NLRP3 may bind directly to ox-mtDNA. While this manuscript was revised, several new articles have been published or are pre-printed highlighting the involvement of mitochondria.

The binding of ox-mtDNA seems to be at least partially overlapping with the region required for binding to PI4P lipids, making it difficult to delineate these two effects and leaving it outside of the scope of this manuscript.

I want to only raise some minor points that are largely on the manuscript and the discussion, and will not require extensive addition of data.

1 The priming step of NLRP3 is mainly depending on TLR-signalling and more specifically subsequent NF-kB activation and not necessarily LPS/TLR4. For reference see DOI: 10.4049/jimmunol.0901363 (line 63/64)

2 In lines 180-190 one should mention the decamer structure of NLRP3 again, highlighting that this region could not be completely resolved by EM and is mostly modelled as loops without a fixed secondary structure.

3 The publications on human and murine NLRP3 decamer formation strongly suggest that the PYD are buried within the cage and are kept inaccessible from interaction with ASC. However, the described positive patch on the NLRP3 surface in this structure is well accessible and hypothesized to allow PI4P binding as a decamer. How do the authors imagine ox-mtDNA would be able to bind here?

4 In the paragraph discussing ROS production in Mitochondria the publication Billingham et al (https://doi.org/10.1038/s41590-022-01185-3) should be mentioned as this highlights that ROS produced in mitochondria is not strictly necessary. (line 278-286)

5 In line 300-301 the authors mention inhibitory interactions between the PYD and LRRs of NLRP3. I am not aware of any interaction between the PYD and LRRs and presume this is supposed to be

NACHT and LRRs (which makes in the context of MCC950).

6 The idea that K2 of the PYD might have an enzymatic function is too far-fetched. The very N-terminal end of PYD is important for NLRP3 activation (see literature on the Y5 phosphorylation) but flexible and not embedded into a fixed enzymatic fold as K249 is in hOGG1. (lines 301-304)

7 How does the PYD of ASC compare to the PYD of NLRP3? Would ox-mtDNA be able to also bind to the PYD of ASC and by this potentially helping interaction and or change in conformation.

Dear Reviewers,

We have prepared a revised version of our paper “Characterization of a Direct Ligand Interaction that Mediates NLRP3 Inflammasome Activation”, considering the comments of the referees. A detailed list of the responses to the comments is appended below.

Reviewer 1.

This reviewer was appreciative of the effort involved in our work and required no additional experiments.

1. *Please insert the reference for the Chandal paper - there is controversy around whether ROS is required for NLRP3 activation and that should be acknowledged rather than ignored.*

We have added references for Chandel 2022 paper, as well as Xian et al, 2023 *Immunity* to note this controversy.

2. *I understand that time and money are limiting but in this case, I'd also appreciate, in the absence of site directed mutagenesis of the DNA binding site or any cell based assay, that these limitations also be included in the discussion.*

These experiments are underway. We mention in the Discussion that we will continue site-directed mutagenesis experiments and validation of all residues interacting with mitochondrial DNA via cryo-EM.

3. *I really appreciate the effort the authors put into looking and annotating the IDR regions of the different NLR proteins, this was interesting and will hopefully help the manuscript.*

We agree and appreciate this comment.

4. *I have some concerns about the specificity of the interaction between the DNA and NLRP3 given that it seems to bind to two different domains. I don't need this addressed but it should be taken into account or dealt with in any follow up work.*

We agree the molecular determinants of recognition need to be addressed for both sites. These experiments are underway.

5. *In response to this comment "that there is no clear reason for the difference in the flow through between the non-oxidised and oxidised DNA". this is really concerning to be honest. I understand that it is a native gel, however all of these are native gels. If the system is not stable enough to give reproducible results between different gels how can the results from this experiment be trusted?*

The Figure 1D referenced is a denaturing gel. All electromobililty shift assays (EMSA’s) shown are native. All DNA competition assay experiments are analyzed with denaturing conditions.

6. *Can units please be added to all figures, they are still missing in figure 2A for example. The concentration units are also missing (uM, nM?) and are the dotted lines the SEM?*

We have updated units for figures. For example, in Figure 2A, we have included that the DNA concentration is in nM and that the dotted lines are the 95% confidence interval of the best fit

line.

7. *The start of the C790T blot for fig 2A is still cut off at the front.*

The format of these blots has been rectified include the full image aligned with the other two.

8. *I still have concerns about figure 2A as there are no controls for:*
- How much NLRP3 comes off the column when you don't have any mtDNA present?
- The total amount of NLRP3 bound to the beads prior to any elution and without this you don't know the proportion of NLRP3 eluting and whether the amount is relevant.

We agree and have corrected this figure and denoted the first lane as 0 nM DNA, which represents a control; the total amount of protein signal on the beads prior to elution.

Reviewer 2.

This reviewer was appreciative of the effort involved in our work and did not require additional experiments.

1. *It would be nice to make minor changes to ambiguous sentences such as:*

Line 112 "there is no binding or that binding has occurred the complex does not enter the gel"

We edited this sentence to clearly denote the two plausible interpretations.

2. *Line 186 "residues known to be important in binding ox-DNA that were exactly the same were"*

We have edited this sentence to communicate conserved residues between NLRP3 and the glycosylase.

3. *Line 194 "Software D2P2 analysis showed several IDR prediction software programs agreed that"*

We edited this sentence to indicate the 100-residue stretch between the pyrin and NACHT domain is predicted to be an intrinsically disordered region.

4. *Line 534 "examined for binding to ether 8-oxo-dGTP or 8-oxo-dG molecule" (ether should be either)*

We have made the suggested correction.

5. *It would also be nice to have information on how different NLRP3 constructs (1-93, 1-134, 94-1034) were generated. Authors described NLRP3 (1-93) mutagenesis in some detail (in the supplementary methods), so it would also be helpful to have information on others.*

Details of how $NLRP3_{1-134}$ and $NLRP3_{94-1034}$ mutants were made are now included in the Supplement. Briefly, $NLRP3_{1-134}$ was made by adding a stop codon after residue 134 using site-directed mutagenesis. The $NLRP3_{94-1034}$ was outsourced to Azenta to delete the region between the N-terminal 6xHis Tag and residue 94.

Reviewer 3.

This reviewer was appreciative of the effort involved in our work and required no additional experiments.

1. *The priming step of NLRP3 is mainly depending on TLR-signaling and more specifically subsequent NF-kB activation and not necessarily LPS/TLR4. For reference see DOI: 10.4049/jimmunol.0901363 (line 63/64).*

We have clarified our description and added the recommended reference.

2. *In lines 180-190 one should mention the decamer structure of NLRP3 again, highlighting that this region could not be completely resolved by EM and is mostly modelled as loops without a fixed secondary structure.*

The reviewer is correct that in the decamer is missing well-resolved density from the pyrin domain to the NACHT for several protomers. We show 1 of 2 full-length protomers that have large unstructured regions in Figure 3C.

3. *The publications on human and murine NLRP3 decamer formation strongly suggest that the PYD are buried within the cage and are kept inaccessible from interaction with ASC. However, the described positive patch on the NLRP3 surface in this structure is well accessible and hypothesized to allow PI4P binding as a decamer. How do the authors imagine ox-mtDNA would be able to bind here?*

We have analyzed solvent accessible regions of the decamer and can measure “pores” up to 24 Å which are sufficient to accommodate entrance of B-DNA.

4. *In the paragraph discussing ROS production in Mitochondria the publication Billingham et al (https://doi.org/10.1038/s41590-022-01185-3) should be mentioned as this highlights that ROS produced in mitochondria is not strictly necessary. (line 278-286).*

The reviewer is correct. We have added this reference, as well as references for a paper from the Chandel group in 2022, as well as Xian et al, 2023 *Immunity* to site this finding.

5. *In line 300-301 the authors mention inhibitory interactions between the PYD and LRRs of NLRP3. I am not aware of any interaction between the PYD and LRRs and presume this is supposed to be NACHT and LRRs (which makes in the context of MCC950).*

The reviewer is correct. We have corrected this sentence to indicate inhibitory interactions between NACHT and LRR in the context of the inhibitor MCC950.

6. *The idea that K2 of the PYD might have an enzymatic function is too far-fetched. The very N-terminal end of PYD is important for NLRP3 activation (see literature on the Y5 phosphorylation) but flexible and not embedded into a fixed enzymatic fold as K249 is in hOGG1. (lines 301-304).*

The reviewer is correct that K249 is structured in hOGG1 and while K2 is unstructured in NLRP3. Our analysis shown in Figure S3 supports that the NLRP3 N-terminus is predicted to be an intrinsically disordered region (IDR). These IDR’s are known to convert from ordered to

disordered depending on if they are interacting with a macromolecular partner/ligand or not. Moreover, although the pyrin domain of NLRP3 and hOGG1 superpose reasonably, the hOGG1 structure is bound to DNA and has a K249Q mutation and NLRP3 is not interacting with anything at the N-terminus where the K2 resides.

7. *How does the PYD of ASC compare to the PYD of NLRP3? Would ox-mtDNA be able to also bind to the PYD of ASC and by this potentially helping interaction and or change in conformation.*

We have not been able to find sequence similarities, nor superpositions that predict ASC can bind DNA. Nonetheless, we will evaluate this empirically in our future experiments.

We hope that with these changes, the paper will be acceptable for publication in *Communications Biology*.

Yours sincerely,

Reginald McNulty.
